# Exploring the transcriptional crosstalk between adipose tissue and locoregional recurrence in breast cancer using independent component analysis

**DOI:** 10.1007/s10142-026-02012-w

**Published:** 2026-09-09

**Authors:** Marlous Arjaans, Bert van der Vegt, Renske Linstra, Rudolf S. N. Fehrmann, Arkajyoti Bhattacharya

**Affiliations:** 1 Department of Plastic Surgery, University Medical Center Groningen, University of Groningen, Groningen, the Netherlands; 2 Department of Plastic Surgery, OLVG Medical Center, Amsterdam, the Netherlands; 3 Department of Pathology and Medical Biology, University Medical Center Groningen, University of Groningen, Groningen, The Netherlands; 4 Department of Medical Oncology, University Medical Center Groningen, University of Groningen, Groningen, the Netherlands

**Keywords:** Breast cancer, Adipose tissue, Locoregional recurrence, Breast reconstructive surgery

## Abstract

Locoregional recurrence (LRR) poses a persistent clinical challenge in breast cancer, with emerging evidence implicating the tumor-associated adipose tissue in modulating recurrence risk. This study investigates shared transcriptional programs between adipose tissue and breast tumors and examines their association with disease-free survival (DFS), particularly in the context of reconstructive surgery where adipose tissue from different body compartments are commonly used. We analyzed bulk gene expression data from 5,691 breast tumors and 978 human adipose tissue samples from different body compartments using consensus-independent component analysis (c-ICA) to identify transcriptional components (TCs). Gene set enrichment analysis (GSEA) and copy number alteration profiling were used for biological annotation. Associations between TCs and DFS were evaluated through univariate Cox regression. Key findings were validated using spatial transcriptomic and single-cell RNA sequencing datasets. Among the 411 TCs identified, 332 showed biological enrichment, and 35 were significantly associated with DFS. Four DFS-associated TCs (TC257, TC350, TC371, TC400) were enriched for adipogenesis-related genes and exhibited heightened activity in high-grade, triple-negative tumors and in patients with elevated BMI. Notably, TC350 was highly active in adipose tissue from common reconstructive donor sites (abdomen, omentum, subcutis) but not in native breast adipose tissue. Spatial transcriptomic and single-cell analyses confirmed the increased activity of these adipogenesis-related TCs in tumor regions and adipose cells. TC350 included *FABP4*, a gene previously linked to poor prognosis in breast cancer and considered as a potential new therapeutic target. Adipose tissue-derived transcriptional programs influence breast cancer prognosis and this seems to differ by tissue origin. These findings generate a hypothesis that donor site selection for adipose tissue in reconstructive surgery may impact LRR risk through adipogenesis-associated mechanisms. Further research is warranted to elucidate the biological and clinical implications of adipose–tumor transcriptional interactions.

**Supplementary Information:**

The online version contains supplementary material available at https://doi.org/10.1007/s10142-026-02012-w.

## Background

Breast cancer remains the leading cause of cancer-related mortality among women in developing countries and ranks second in developed nations (Society AC 2015) Despite significant therapeutic progress, locoregional recurrences (LRR) persists as a major clinical challenge, with 5-year survival rates ranging from 40 to 65% (Zimmerman et al. 2014; Clemons et al. 2001; Tienhoven et al. 1999; Voogd et al. 2005). This highlights the urgent need to identify factors contributing to elevated LRR risk.

Recent studies highlight the tumor microenvironment’s role in LRR, particularly the influence of adipose tissue (Hanahan and Weinberg 2011; Hanahan and Coussens 2012; Ostman 2012). Breast adipose tissue is predominately composed of mature adipocytes (~ 90%), with the remaining stromal-vascular fraction (SVF) contributing ~ 10% (Wronska and Kmiec 2012). Once considered merely an energy reservoir, adipocytes can transition into cancer-associated adipocytes (CAAs) in the presence of tumor cells, promoting proliferation, migration, and invasiveness in preclinical models (Zhao et al. 2020; Dirat et al. 2011). Additionally, genetic susceptibility factors such as breast cancer gene 1 (BRCA1) mutations may alter adipose-derived stem cells, driving more aggressive cancer behavior (Zhao et al. 2019). While direct clinical evidence linking adipose tissue to LRR is limited, findings from a phase III trial of nearly 5,000 patients suggest that obese individuals with hormone-receptor-positive disease have an increased risk of LRR between 3- and 8-years post-diagnosis, whereas obese patients with triple-negative breast cancer (TNBC) appear to have a lower risk during the first three years (Sparano et al. 2015). These observations underscore the complex interplay between obesity and LRR, emphasizing the need for a more nuanced understanding of adipose tissue’s role.

With the increasing prevalence of breast reconstruction surgeries involving adipose tissue, understanding adipocytes’ impact on LRR is becoming even more crucial. Autologous reconstruction using adipofasciocutaneous flaps is the gold standard, with common donor sites including the abdomen, thigh, lower back, buttocks, and omentum (Feng et al. 1994; Patel and Ramakrishnan 2017; Tuinder et al. 2018; Zaha et al. 2006). However, lipofilling, which involves harvesting adipose tissue via liposuction for defect correction, has raised concerns regarding oncological safety. Specifically, harvested adipocytes stem cells may stimulate residual tumor cells and promote recurrence; however, the current literature—largely retrospective cohort studies—remains inconclusive (Lohsiriwat et al. 2011; Krastev et al. 2013).

The availability of bulk gene expression profiles from both breast cancer patients and adipose tissues offers a valuable opportunity to explore the role of adipose tissue in LRR risk. However, the bulk profiles represent an aggregate of tumor cells and various components of the tumor microenvironment, which can obscure subtle adipocyte-specific transcriptional signals pertinent to LRR. To address this limitation, we applied consensus-independent component analysis (c-ICA) to decompose bulk gene expression profiles into statistically independent transcriptional components (TCs), each reflecting distinct biological processes (Urzúa-Traslaviña et al. 2021). This method enables the identification of both dominant and more subtle TCs and allows for their activity to be quantified in individual samples.

Previous studies have shown that transcriptomic studies can aid in defining biological relevant processes and even identify possible new biomarker (Ebrahim et al. 2025a, 2025b, 2025c, 2026).

In this transcriptomic hypothesis-generating study, we investigated whether adipose tissue from different donor sites exhibits distinct transcriptional patterns and examined whether these patterns are associated with LRR in breast cancer patients. We compiled bulk gene expression profiles from non-metastatic breast tumor samples and human adipose tissue samples, used c-ICA and Gene Set Enrichment Analysis (GSEA) to identify adipose tissue-specific TCs, and subsequently assessed the association of these TCs with disease free survival (DFS). Finally, we evaluated the spatial co-localization of DFS-associated TCs in spatially resolved transcriptomic data.

## Methods

**Fig. 1:**
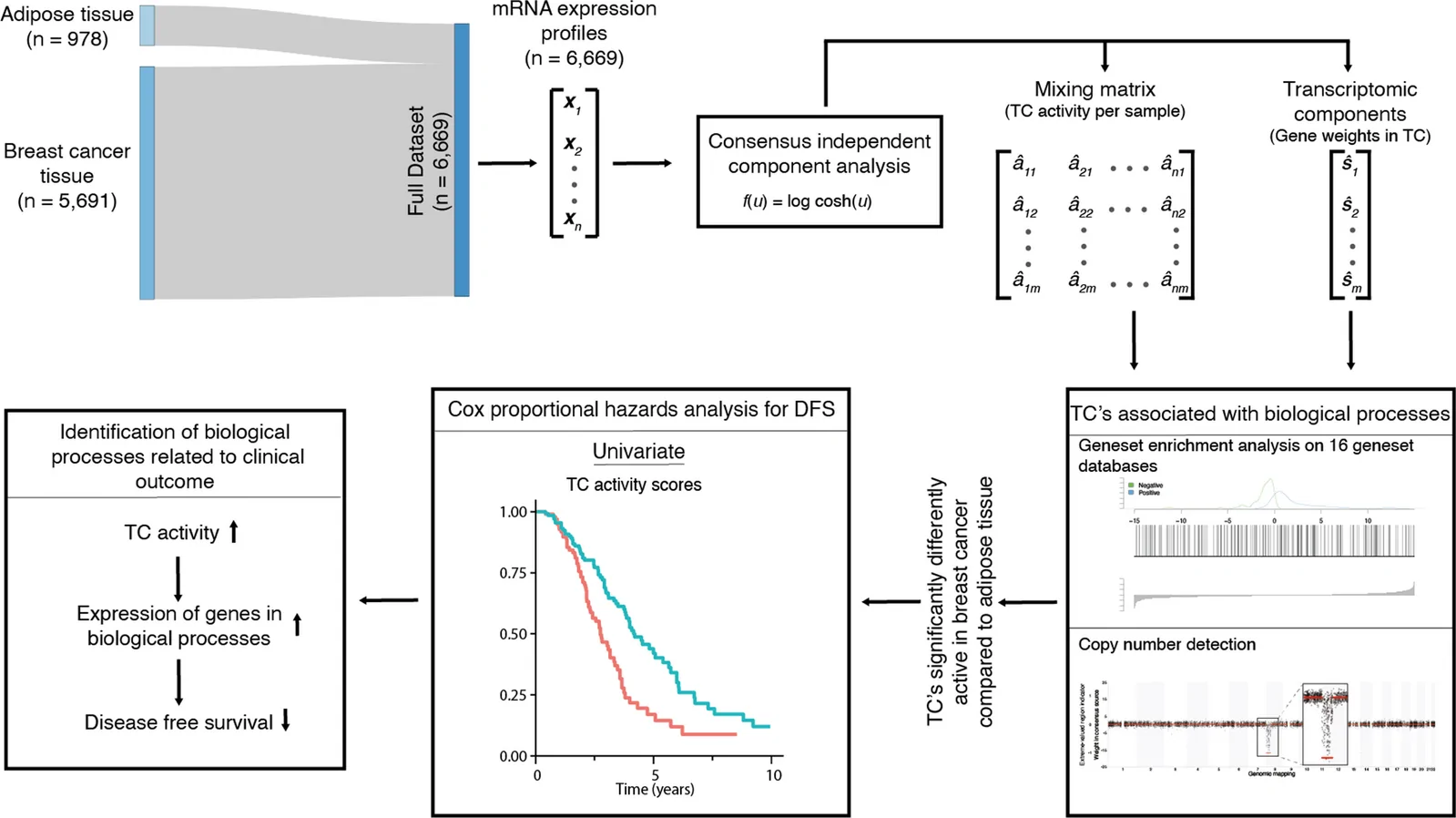
Workflow indicating relations between the methods

A detailed description is provided in the Additional Methods and in Fig. 1 an overview is presented.

### Data acquisition, preprocessing, and quality control

Data acquisition, preprocessing, and quality control were performed as described previously (Urzúa-Traslaviña et al. 2021; Clough and Barrett 2016; Fehrmann et al. 2015). Raw microarray bulk gene expression profiles of breast cancer samples and adipose tissues of non-cancer patients were obtained from the Gene Expression Omnibus (GEO) (Fehrmann et al. 2015). Additionally, clinicopathological information and follow-up data was obtained. Only samples processed using the Affymetrix HG-U133 Plus 2.0 platform (GPL570) were included, while those derived from cell lines or animal models were excluded. Duplicate samples were identified and removed based on MD5 hashes and high Spearman correlation coefficients (*R* > 0.99). The robust multiarray algorithm was applied to preprocess and aggregate raw data, followed by principal component analysis for quality control, as previously described (Fehrmann et al. 2015).

We additionally retrieved 11 spatially resolved transcriptomes from breast cancer patients through Zenodo and the 10 × Genomics repository (see Additional methods). Moreover, 10,000 single-cell profiles from adipose tissue of a healthy participant were obtained from GEO (study ID GSE134355). Genes with no detectable expression in all samples were excluded. For both the spatially resolved and single-cell transcriptomes, mRNA expression levels were normalized by removing the first principal component derived from the sample-wise correlation matrix.

### Clinicopathological data collection

Clinicopathological data extracted for the breast cancer profiles included sex, age, tumor histological subtype, tumor grade, tumor size, TNM stage, lymph node involvement, estrogen receptor (ER), progesterone receptor (PR) and human epidermal growth factor receptor 2 (HER2) status, BRCA1/2 mutation status, Ki67 proliferation index, menopausal status, body mass index (BMI), breast cancer subtype (both receptor based and intrinsic), treatment regimen, and DFS. Receptor status was included only when assessed according to the immunohistochemistry staining guidelines of the American Society of Clinical Oncology and College of American Pathologists (Hammond et al. 2010; Wolff et al. 2013; Coates et al. 2015). BRCA1/2 mutation were further classified as either germline or somatic. DFS was defined as the interval from diagnosis until disease recurrence, death, or last follow up.

For human adipose tissue profiles, we collected data on tissue’s anatomical origin, sex, age, BMI (categorized in accordance WITH World Health Organization definitions), and menopausal status. Missing data were designated as “Not available”.

### Consensus independent component analysis (c-ICA)

We applied c-ICA to the preprocessed gene expression data, decomposing the profiles into statistically independent transcriptional signatures, referred to as TCs (Chiappetta et al. 2004; Metsalu and Vilo 2015). Each TC represents a distinct biological process, and the weight assigned to each gene indicates the direction and magnitude of that process’s influence on the gene’s expression. In addition to identifying TCs, c-ICA also generated a mixing matrix, which provides the activity scores of each TC for individual samples.

### Biological characterization of transcriptional components

We employed several approaches to characterize the identified TCs. First, we performed GSEA using 15 gene set collections from the Molecular Signatures Database (MsigDB) version 7.1 (see additional methods for details) (Chiappetta et al. 2004). A TC was deemed significantly enriched for a given gene set if the absolute z-score exceeded 3. For visualization, we used the web-based tool ClustVis (Metsalu and Vilo 2015) to generate enrichment heatmaps, focusing on gene sets whose enrichment scores surpassed the Bonferroni-adjusted threshold for at least one TC. Second, we applied the Transcriptional Adaptation to Copy Number Alterations (TACNA) profiling technique (Bhattacharya et al. 2020) to identify TCs that capture the downstream effects of copy number alterations (CNAs) on gene expression levels.

### Univariate survival analysis

We conducted univariate Cox proportional hazards regression on a subset of breast cancer patients for whom DFS data were available, aiming to evaluate the association between TC activity and DFS. To limit false positives resulting from multiple testing, we employed a permutation-based framework with a false discovery rate (FDR) threshold of 1% and a confidence level (CL) of 80%.

### Identification of significantly active TCs in individual gene expression profiles

We calculated the activity score of each TC in a given gene expression profile by taking the dot product of the pseudo-inverse-derived TC vector with the corresponding expression levels in that profile. To determine the significance of this activity score, we used a permutation-based methodology: for each profile-TC pair, we performed 5,000 permutations of the gene weights within the TC and repeated activity score calculation, thus generating a null distribution of activity scores. If the Anderson–Darling test indicated that this null distribution deviated from normality, we applied a Johnson transformation— using one of three optimal families of distributions (S, SU, SL)—to both the null distribution and the observed activity score. Afterward, we standardized the transformed null distribution to have a mean of zero and a standard deviation of one, applying the same standardization to the observed activity score. We then fit symmetrical generalized Gaussian distributions to these null distributions to derive p-values for each profile-TC pair. A TC was deemed significantly active if its p-value was below 0.01. For each tissue compartment, we calculated the proportion of samples exhibiting significant activity for each TC, separately for positive and negative activity scores. In analyses involving spatially resolved and single-cell transcriptomes, we further used z-transformed p-values for data visualization.

### Calculation of credibility index of DFS associated TCs and reproducibility

To evaluate the stability of the identified TCs across independent ICA initializations, we repeated ICA 96 times. Independent components generated across these runs were clustered based on similarity, and consensus TCs were derived from these clusters. For each consensus TC, we calculated a credibility index by dividing the number of independent components included in the corresponding cluster by the total number of ICA runs. A higher credibility index therefore indicates that the same transcriptional pattern was repeatedly identified across independent ICA runs and is less likely to represent a run-specific artifact.

To evaluate whether the DFS-associated TCs could be detected in independent transcriptomic resources, we projected the 35 DFS-associated TC gene-weight vectors onto external breast cancer transcriptomes from The Cancer Genome Atlas (TCGA) and breast cancer cell-line profiles from the Cancer Cell Line Encyclopedia (CCLE). Projection was performed conceptually as described previously for cross-dataset TC analyses in colorectal cancer and ovarian cancer (Knapen et al. 2024; Bhattacharya et al. 2025). Briefly, expression profiles in the external datasets were aligned to the genes represented in the TC matrix, and TC activity scores were calculated for each external sample using the pseudo-inverse-derived TC vectors and the corresponding expression levels. Activity distributions were then summarized using box-and-whisker and dot plots for TCGA breast carcinoma subgroups and for CCLE breast cancer cell lines. Outlier activity was visualized using thresholds of approximately ± 3 activity-score units, indicating samples or cell lines in which the transcriptional program captured by a TC was particularly active or inactive relative to the projected distribution.

### Multivariate Cox regression model

To assess whether the association between DFS-associated TCs and DFS was independent of established clinicopathological variables, we performed multivariate Cox proportional hazards regression. Separate models were fitted for each of the 35 DFS-associated TCs. DFS time in months and DFS event status were used as the survival outcome, and the activity score of the individual TC was entered as the predictor of interest. Models were adjusted for intrinsic molecular subtype, tumor grade, and lymph node involvement, which were selected as clinically established prognostic variables with sufficient availability in the integrated public cohorts. Hazard ratios (HRs), Wald 95% confidence intervals (CIs), z statistics, and P values were extracted for the TC activity term. Given the retrospective nature of the integrated datasets and incomplete annotation across all studies, variables such as BMI, BRCA mutation status, and treatment regimen were not included in the final multivariate model to avoid excessive loss of samples and unstable model estimates.

## Results

### Dataset characteristics of breast cancer and adipose tissue samples

**Fig. 2:**
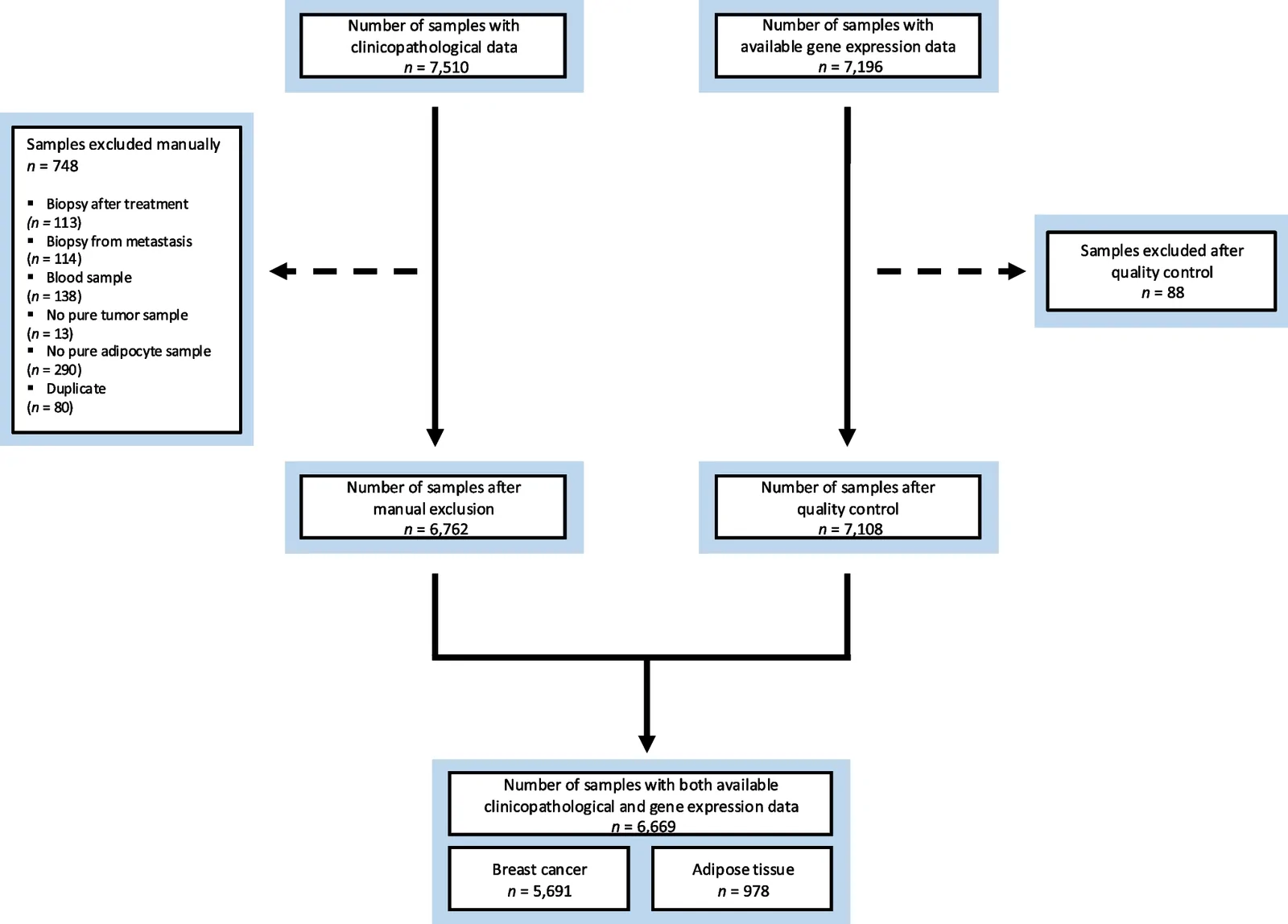
Flowchart of data acquisition

We compiled a comprehensive dataset of 6,083 breast cancer and 1,427 adipose tissue bulk gene expression profiles from GEO. Following preprocessing, duplicate removal, and quality control, the final dataset included 5,691 breast cancer samples and 978 adipose tissue samples, derived from 99 breast cancer studies (73 unique GEO series) and 25 adipose tissue studies (19 unique GEO series) (See Fig. 2, Additional Tables 1 and 2). Detailed clinicopathological information for both sample types is summarized in Tables 1 and 2. Adipose tissue samples originated from eight anatomical sites: breast (*n* = 113), abdomen (*n* = 616), omentum (*n* = 41), pericard (*n* = 26), parathyroid gland (*n* = 9), visceral (*n* = 66), subcutaneous (*n* = 105), and unknown origin (*n* = 2). Samples from unknown sites were excluded from further analyses.

**Table 1:** Patient characteristics of the breast tumor tissue samples

	Provided samples (5,691) *N* (%)	Inferred samples (5,691) *N* (%)
Sex		
Female	5,691 (100.00)	
Male	0 (0)	
Age in years		
21–30	50 (0.88)	
31–40	364 (6.40)	
41–50	790 (13.88)	
51–60	792 (13.92)	
61–70	520 (9.14)	
71–80	241 (4.23)	
81–90	45 (0.79)	
NA	2,889 (50.76)	
Molecular subtype (IHC)		
Luminal A	47 (0.83)	1,529 (26.87)
Luminal B	61 (1.07)	2,072 (36.41)
HER2 enriched	72 (1.27)	572 (10.05)
Basal like	397 (6.98)	1,518 (26.67)
NA	5,114 (89.86)	0 (0.00)
Molecular subtype (Intrinsic)		
Luminal A	185 (3.25)	1,959 (34.42)
Luminal B	140 (2.46)	2,096 (36.83)
HER2 enriched	99 (1.74)	340 (5.97)
Basal like	225 (3.95)	1,189 (20.89)
Normal like	39 (0.69)	107 (1.88)
NA	5,003 (87.91)	0 (0.00)
BRCA 1/2 mutation		
BRCA1 somatic mutation	34 (0.60)	656 (11.53)
BRCA2 somatic mutation	6 (0.11)	6 (0.11)
Germline mutation	15 (0.26)	56 (0.98)
Wild type	139 (2.44)	4,973 (87.38)
NA	5,497 (96.59)	0 (0.00)
Tumor histological subtype		
DCIS	22 (0.39)	
IDC	1,043 (18.33)	
ILC	193 (3.39)	
Mixed	36 (0.63)	
Other	48 (0.84)	
NA	4349 (76.42)	
Tumor grade		
1	262 (4.60)	398 (6.99)
2	818 (14.37)	1,957 (34.39)
3	1,236 (21.72)	3,336 (58.62)
NA	3,375 (59.30)	0 (0.00)
Tumor size (cm)		
size < = 2	408 (7.17)	
2 < size < = 5	512 (9.00)	
size > 5	50 (0.88)	
NA	4,721(82.96)	
T—stage		
T1	548 (9.63)	
T2	1,103 (19.38)	
T3	177 (3.11)	
T4	59 (1.04)	
NA	3,804 (66.84)	
N—stage		
N0	713 (12.53)	
N1	482 (8.47)	
N2	196 (3.44)	
N3	146 (2.57)	
NA	4154 (72.99)	
M—stage		
M1	111 (1.95)	111 (1.95)
M0	1,093 (19.21)	5,580 (98.05)
NA	4487 (78.84)	0 (0.00)
Lymph node involvement		
Positive	1,592 (27.97)	
Negative	1,336 (23.48)	
NA	2,763 (48.55)	
ER status		
Positive	1,849 (32.49)	3,537 (62.15)
Negative	1,204 (21.16)	2,154 (37.85)
NA	2,638 (46.35)	0 (0.00)
PR status		
Positive	1,072 (18.84)	2,944 (51.73)
Negative	1,097 (19.28)	2,747 (48.27)
NA	3,522 (61.89)	0 (0.00)
HER2 status		
Positive	652 (11.46)	1,289 (22.65)
Negative	1,902 (33.42)	4,402 (77.35)
NA	3,137 (55.12)	0 (0.00)
BMI		
Underweight	4 (0.07)	
Normal weight	63 (1.11)	
Pre-obesity	86 (1.51)	
Obesity class I	47 (0.83)	
Obesity class II	17 (0.30)	
Obesity class III	7 (0.12)	
NA	5,467 (96.06)	
(Neo)Adjuvant therapy		
No treatment	142 (2.50)	
Hormone therapy	157 (2.76)	
Chemotherapy	697 (12.25)	
Anti-HER2	94 (1.65)	
Hormone therapy + Chemotherapy	6 (0.11)	
AntiHER2 + Chemotherapy	107 (1.88)	
Radiotherapy + Chemotherapy	28 (0.49)	
Radiotherapy + Hormone therapy	29 (0.51)	
Radiotherapy + Hormone therapy + Chemotherapy	19 (0.33)	
NA	4,412 (77.53)	
Disease Free Survival		
Events recurrence	120 (2.11)	
Events censored	279 (4.90)	
Median follow up time in months/range	73.37/0.00—222.29	
NA	5,292 (92.99)	
Menopausal status		
Pre	209 (3.67)	797 (14.00)
Post	474 (8.33)	4,894 (86.00)
NA	5,008 (88.00)	0 (0.00)
Ki67 score		
< = 20%	218 (3.83)	1,827 (32.10)
> 20%	269 (4.73)	3,864 (67.90)
NA	5,204 (91.44)	0 (0.00)

*NA* Not available

**Table 2:** Patient characteristics of the adipose tissue samples

	Samples provided (total = 978) *N* (%)
Sex	
Female	508 (51.94)
Male	249 (25.46)
Not specified	66 (6.75)
NA	155 (15.85)
Age in years	
0–10	8 (0.82)
11–20	2 (0.20)
21–30	4 (0.41)
31–40	7 (0.72)
41–50	23 (2.35)
51–60	60 (6.13)
61–70	5 (0.51)
71–80	1 (0.10)
NA	868 (88.75)
BMI	
Underweight	1(0.10)
Normal weight	5 (0.51)
Pre-obesity	49 (5.01)
Obesity class I	20 (2.04)
Obesity class II	17 (1.74)
Obesity class III	30 (3.07)
NA	856 (87.53)
Menopausal status	
Pre	0 (0.00)
Post	70 (7.16)
NA	908 (92.84)
Origin adipose tissue	
Abdomen	616 (62.99)
Breast	113 (11.55)
Subcutaneous tissue	105 (10.74)
Visceral	66 (6.75)
Omentum	41 (4.19)
Pericardium	26 (2.66)
Parathyroid gland	9 (0.92)
NA	2 (0.20)

*NA* Not available

### Identification of 332 biologically enriched transcriptional components with c-ICA

We applied c-ICA to uncover transcriptional signatures of biological processes, identifying 411 statistically independent TCs. Of these, 245 reflected the transcriptional effects of CNAs (Table 3), as determined by TACNA profiling. An additional 87 TCs were significantly enriched for at least one gene set from the 15 collections in MsigDB, bringing the total to 332 biologically enriched TCs (Table 4).

**Table 3:** Characteristics of copy number alterations TCs

TC	Number of genes in extreme valued region for each chromosome																								Total number of genes
	1	2	3	4	5	6	7	8	9	10	11	12	13	14	15	16	17	18	19	20	21	22	X	Y	
TC 11																10									10
TC 368											10														10
TC 307		11																							11
TC 84																								12	12
TC 278											12														12
TC 330											13														13
TC 124		14																							14
TC 172					14																				14
TC 194																	14								14
TC 251																	15								15
TC 153					16																				16
TC 391																	16								16
TC 139																			17						17
TC 279																			17						17
TC 75											18														18
TC 104																	18								18
TC 334																	18								18
TC 101							19																		19
TC 173																	19								19
TC 176																	19								19
TC 196																	20								20
TC 157						21																			21
TC 192																	22								22
TC 293											22														22
TC 326																	22								22
TC 229							23																		23
TC 224																	25								25
TC 283											26														26
TC 149																	28								28
TC 213							28																		28
TC 318																	28								28
TC 320						28																			28
TC 79																	30								30
TC 161											31														31
TC 230								31																	31
TC 88										32															32
TC 107											32														32
TC 130											33														33
TC 197																33									33
TC 218																				33					33
TC 321																	34								34
TC 29																				36					36
TC 103																			36						36
TC 348																			36						36
TC 57																	37								37
TC 186																	37								37
TC 362																			37						37
TC 81											40														40
TC 2				41																					41
TC 331											43														43
TC 67														44											44
TC 269												45													45
TC 303															45										45
TC 46																	46								46
TC 108										46															46
TC 270																			46						46
TC 298																	46								46
TC 357												46													46
TC 387										46															46
TC 86								47																	47
TC 228										47															47
TC 234						47																			47
TC 58						49																			49
TC 204													50												50
TC 83												51													51
TC 299																	51								51
TC 59																						52			52
TC 167									52																52
TC 183																				52					52
TC 294								52																	52
TC 225								53																	53
TC 267								53																	53
TC 200											56														56
TC 304											56														56
TC 382																							56		56
TC 142	57																								57
TC 295																				57					57
TC 55			58																						58
TC 73																		58							58
TC 100														58											58
TC 115																				58					58
TC 96																			59						59
TC 119											59														59
TC 182											60														60
TC 305											60														60
TC 42												61													61
TC 193	61																								61
TC 53				63																					63
TC 112											63														63
TC 140			63																						63
TC 248					63																				63
TC 338								63																	63
TC 98											64														64
TC 181																			64						64
TC 271																65									65
TC 191																				69					69
TC 341																	70								70
TC 281										71															71
TC 327																		71							71
TC 28																72									72
TC 174						73																			73
TC 74																			74						74
TC 27										75															75
TC 397																			75						75
TC 317								76																	76
TC 374																			76						76
TC 102		77																							77
TC 141				78																					78
TC 150					78																				78
TC 198						78																			78
TC 188										79															79
TC 233								79																	79
TC 395																					79				79
TC 231										80															80
TC 254														80											80
TC 50																						83			83
TC 76	83																								83
TC 94			83																						83
TC 180						83																			83
TC 44																			84						84
TC 133							84																		84
TC 342					84																				84
TC 113											85														85
TC 329											85														85
TC 405																							87		87
TC 66		88																							88
TC 324																		88							88
TC 17	90																								90
TC 123							90																		90
TC 328																		90							90
TC 146														1									90		91
TC 189	92																								92
TC 261															92										92
TC 386								92																	92
TC 63																			93						93
TC 68				93																					93
TC 90													94												94
TC 111												94													94
TC 129							94																		94
TC 185																			94						94
TC 125			95																						95
TC 375								95																	95
TC 109		96																							96
TC 284									96																96
TC 406										96															96
TC 195							97																		97
TC 214																							97		97
TC 227	97																								97
TC 241	97																								97
TC 243			97																						97
TC 308																				97					97
TC 138				99																					99
TC 201									99																99
TC 132	101																								101
TC 268																			101						101
TC 360												101													101
TC 373														101											101
TC 238	102																								102
TC 34																			103						103
TC 92																	104								104
TC 162					104																				104
TC 135										105															105
TC 203						105																			105
TC 154																							106		106
TC 292																						107			107
TC 394															108										108
TC 30																				109					109
TC 56																			109						109
TC 219												109													109
TC 19															110										110
TC 54																110									110
TC 52						111																			111
TC 187																			111						111
TC 252																	111								111
TC 300																		111							111
TC 6																					112				112
TC 370																	112								112
TC 69															113										113
TC 116	114																								114
TC 240																							114		114
TC 143						116																			116
TC 255	116																								116
TC 265							116																		116
TC 35		117																							117
TC 145													117												117
TC 389			117																						117
TC 367																						118			118
TC 36	120																								120
TC 71																			120						120
TC 80														120											120
TC 274												120													120
TC 378												120													120
TC 222			122																						122
TC 236								123																	123
TC 242																123									123
TC 170				124																					124
TC 349												129													129
TC 276												131													131
TC 23																133									133
TC 99									134																134
TC 380																134									134
TC 134											136														136
TC 155			136																						136
TC 51														139											139
TC 217			140																						140
TC 369																						140			140
TC 205													141												141
TC 250														143											143
TC 209															144										144
TC 206	145																								145
TC 160	146																								146
TC 60																147									147
TC 202														149											149
TC 262									150																150
TC 78																			153						153
TC 114															154										154
TC 207				157																					157
TC 286	157																								157
TC 210												160													160
TC 178											161														161
TC 105																	162								162
TC 404		162																							162
TC 47	165																								165
TC 128																166									166
TC 232									167																167
TC 291					167																				167
TC 82		168																							168
TC 163	169																								169
TC 260																						169			169
TC 355	170																								170
TC 216																							177		177
TC 144							181																		181
TC 126						183																			183
TC 175					183																				183
TC 118												184													184
TC 247							189																		189
TC 169									191																191
TC 223			191																						191
TC 165		194																							194
TC 332					195																				195
TC 381	195																								195
TC 127		203																							203
TC 147										205															205
TC 72		207																							207
TC 61			220																						220

**Table 4:** TCs not capturing effect of copy number alteration and enrichment score above Bonferroni corrected threshold

TC	Maximum enrichment score
TC 179	18,5713
TC 39	16,1594
TC 259	14,6489
TC 14	14,3722
TC 1	13,3054
TC 106	13,1567
TC 26	13,154
TC 272	12,6178
TC 95	12,0982
TC 48	11,7364
TC 137	11,7238
TC 235	11,6626
TC 15	11,3197
TC 65	10,9691
TC 13	10,8541
TC 301	10,6074
TC 4	10,4854
TC 89	10,3728
TC 220	10,372
TC 244	10,2134
TC 18	10,1743
TC 302	9,92192
TC 315	9,87039
TC 166	9,74199
TC 371	9,4633
TC 168	9,37375
TC 152	9,26517
TC 245	9,13608
TC 263	9,10884
TC 296	9,04494
TC 3	8,96657
TC 364	8,96597
TC 212	8,88753
TC 37	8,84051
TC 120	8,72606
TC 314	8,72341
TC 246	8,63747
TC 310	8,36778
TC 237	8,23424
TC 273	8,1167
TC 22	8,06705
TC 171	7,97131
TC 297	7,8838
TC 249	7,85138
TC 358	7,82819
TC 45	7,78023
TC 41	7,74855
TC 158	7,70934
TC 49	7,65509
TC 184	7,63996
TC 393	7,60179
TC 396	7,5131
TC 87	7,51125
TC 344	7,50429
TC 400	7,49823
TC 401	7,33484
TC 350	7,27542
TC 288	7,2202
TC 62	7,16419
TC 7	7,06408
TC 40	7,04966
TC 8	7,03639
TC 117	7,03014
TC 285	6,98395
TC 257	6,86179
TC 199	6,66557
TC 409	6,65945
TC 280	6,63155
TC 151	6,62098
TC 93	6,58075
TC 384	6,57088
TC 309	6,43255
TC 24	6,39584
TC 77	6,39545
TC 408	6,38709
TC 385	6,35949
TC 289	6,31232
TC 110	6,14364
TC 377	5,96976
TC 164	5,96233
TC 346	5,93033
TC 131	5,87961
TC 190	5,8592
TC 177	5,79046
TC 70	5,76551
TC 148	5,76171
TC 215	5,7187

**Fig. 3:**
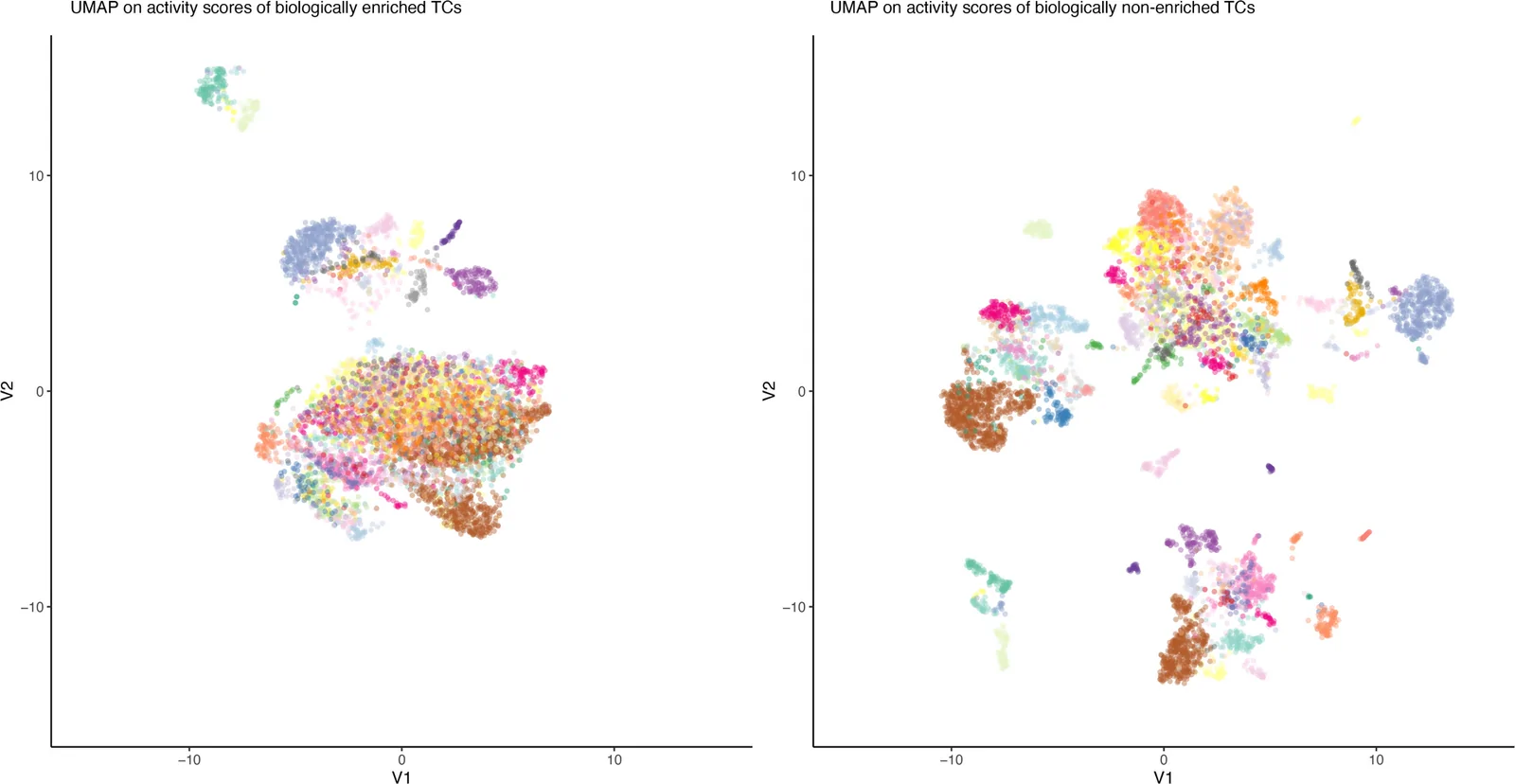
Comparative UMAP distribution of gene expression profiles based on biologically enriched vs. non-enriched TCs. The figure features two panels, each representing a scatterplot of UMAP dimension weights for the samples. The left panel displays the distribution based on the activity scores of biologically enriched transcriptional components (TCs), while the right panel illustrates the distribution based on the activity scores of biologically non-enriched TCs. Biological enrichment is defined as having an enrichment score that surpasses the Bonferroni threshold for multiple testing correction. Each dot in the scatterplots is color-coded according to one of 92 unique series IDs (GSE ID) to demonstrate the distribution of samples from different studies

To compare the 332 biologically enriched TCs with the remaining 79 non-enriched TCs (Additional Table 3), we employed Uniform Manifold Approximation and Projection (UMAP) to visualize their activity scores. As illustrated in Fig. 3, the UMAP plot based on the enriched TCs exhibited markedly less clustering by study ID compared to the plot derived from the non-enriched TCs. This qualitative observation was corroborated by quantitative assessment using the sum of squares of error (SSE) for clustering by study ID (6,453.48 vs. 165.47). A permutation-based test confirmed that this difference was highly significant (*p*-value < 5.0 × 10^–5^). These findings suggest that the 79 non-enriched TCs primarily represent non-biological batch effects. Accordingly, only the 332 biologically enriched TCs were used in subsequent analyses. Comprehensive information on TC composition and GSEA results is available in our public repository: http://transcriptional-landscape-breastcancer-adiposetissue.opendatainscience.net.

### Anatomical origin of adipose tissue associates with activity of biologically enriched TCs

We investigated whether the activity of biologically enriched TCs varies among adipose tissue from different anatomical locations. After applying Bonferroni correction for multiple testing (*p* < 0.01), 331 of the 332 biologically enriched TCs displayed significant differences in activity scores across distinct anatomical sites (Additional Table 4). Furthermore, 292 of these 332 TCs showed significantly different activity scores when comparing adipose tissue and breast cancer samples (Additional Table 5). These finding indicate that the biological characteristics of adipose tissue vary considerably based on anatomical origin.

### Activity scores of 35 biologically enriched TCs are associated with disease free survival in breast cancer patients

**Fig. 4:**
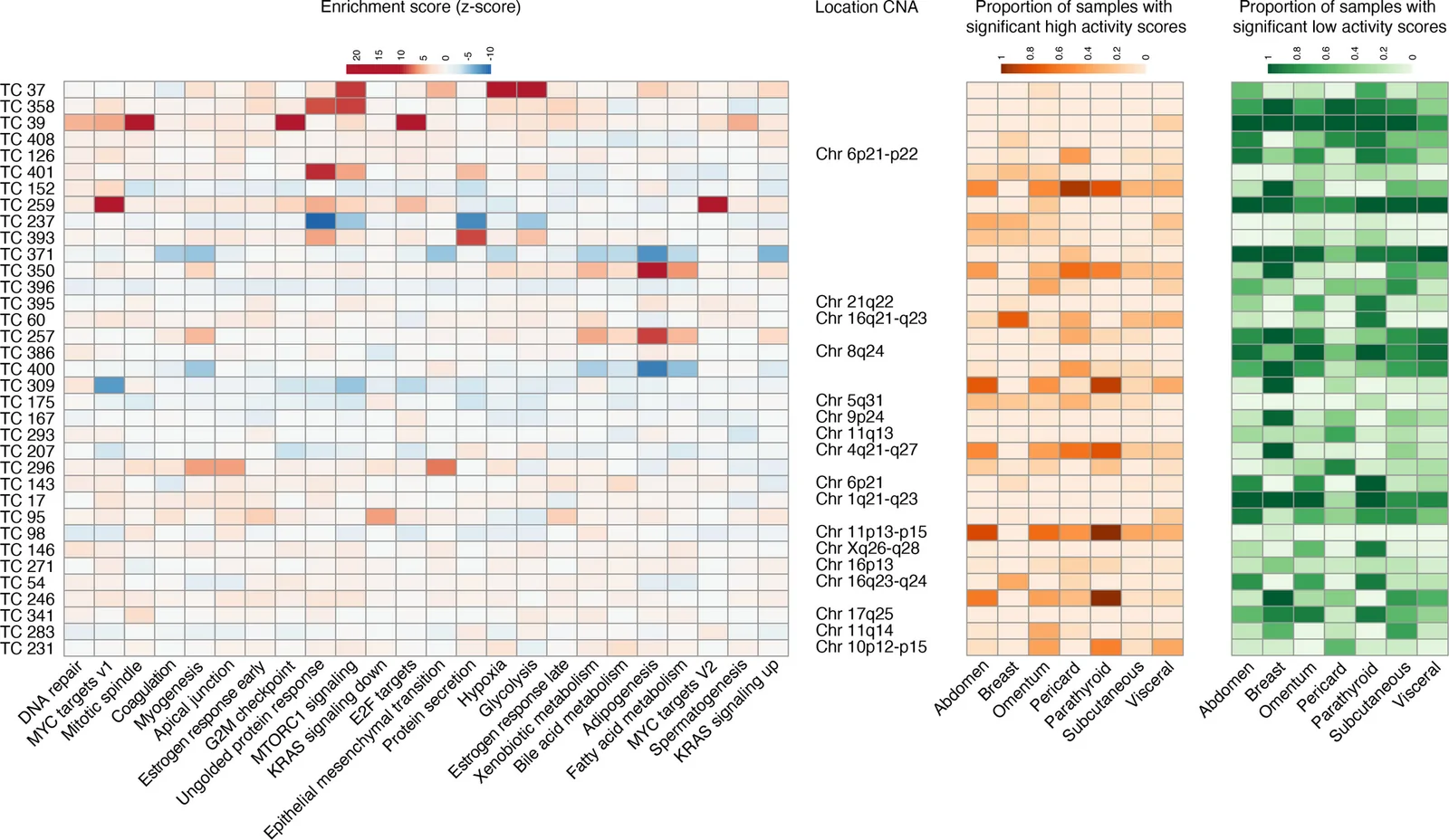
Heatmap depicting hallmark gene set enrichment in DFS-associated TCs and activity levels across adipose tissue. The figure consists of three panels. The leftmost panel showcases GSEA results for the 35 TCs found to be associated with disease-free survival (DFS) in univariate analysis. Included are Hallmark gene sets that meet or exceed the Bonferroni threshold for multiple testing correction. Clustering of gene sets is performed using Pearson correlation and the Ward D2 method, with heatmap colors based on z-scores capped at an absolute value of ten. The middle panel illustrates the proportion of samples showing significantly high activity in each adipose tissue compartment, while the rightmost panel presents the proportion of samples with significantly low activity in these compartments. Proportions range from zero to one

To investigate the relationship between TC activity scores and DFS, we performed univariate Cox proportional hazards regression analysis on a subset of 619 breast cancer patients with available DFS data. Of the 332 biologically enriched TCs, 35 demonstrated a significant association with DFS; these are referred to as DFS-associated TCs. Notably, 31 of these 35 DFS-associated TCs showed significantly different activity between adipose tissues and breast cancer tissues (Bonferroni-adjusted *p*-value < 0.01; Fig. 4 and Additional Table 6).

**Fig. 5:**
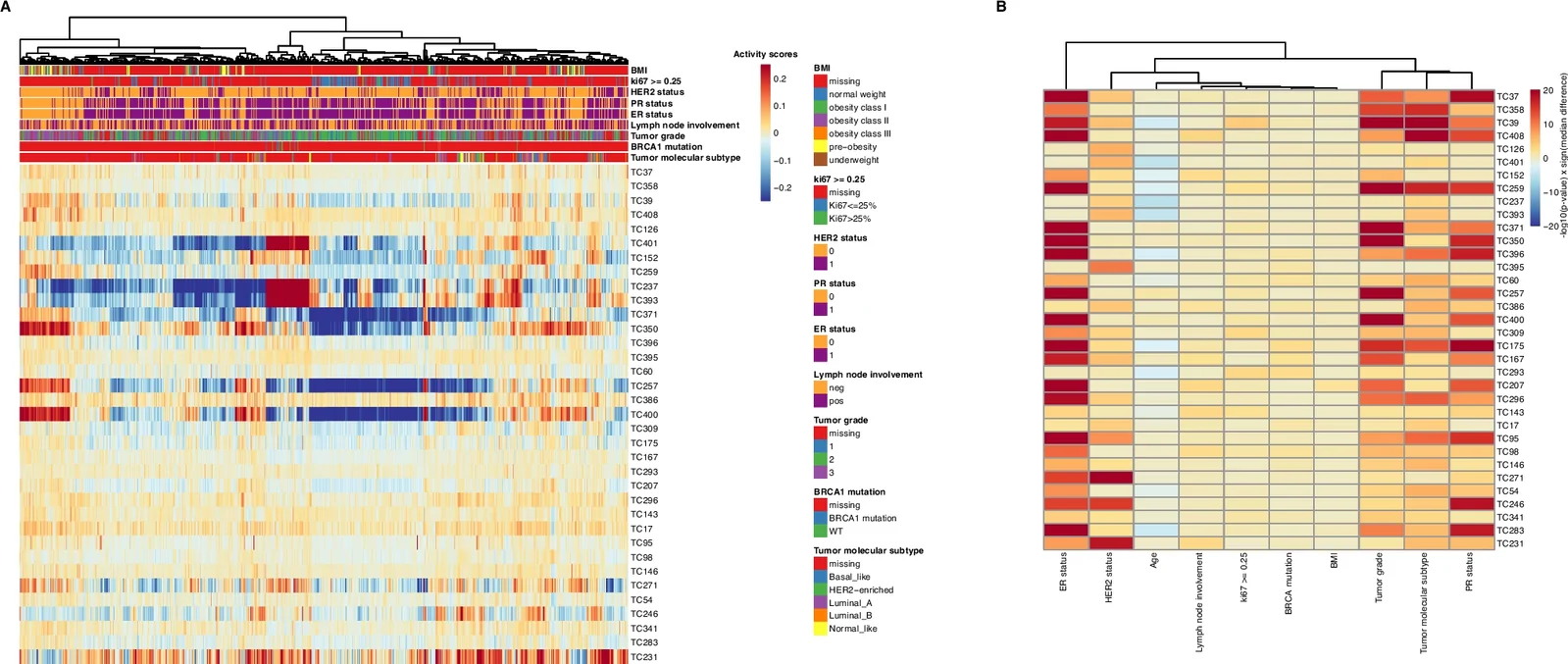
Heatmap depicting activity scores of DFS-associated TCs and association with clinic-pathological variables. **A.** The figure consists of two panels. The top panel showcases the different categories of clinico-pathological variables (BMI, ki67, HER2 status, PR status, ER status, lymph node involvement, tumor grade, BRCA1 mutation status) per sample. The bottom panel showcases activity scores of the 35 TCs found to be associated with disease-free survival (DFS) in univariate analysis. Clustering of samples was performed using Euclidean distance and the complete method, with heatmap colors based on activity scores. **B.** The heatmap showcases the association of activity scores of the 35 TCs found to be associated with disease-free survival (DFS) in univariate analysis with different categories of clinico-pathological variables (BMI, ki67, HER2 status, PR status, ER status, lymph node involvement, tumor grade, BRCA1 mutation status). For categorical variables, -log_10_(p-value)*sign(difference between medians of two grouprs) from Mann–whitney U test was utilized and if the variable is numeric, -log_10_(p-value)*sign(estimate) from correlation test was utilized. Clustering of association score was performed using Euclidean distance and the complete method, with heatmap colors based on association scores

All 35 DFS-associated TCs showed significant enrichment for at least one gene set from the Gene Ontology Biological Processes collection, with a median top z-score of 2.828 (interquartile range of 1.901 to 3.133). Among these, TC37 showed the strongest association with DFS and was highly enriched for genes related to hypoxia, glycolysis and MTORC1 signaling. We identified four TCs (TC257, TC350, TC371, and TC400) enriched for adipogenesis-related genes (referred to as adipogenesis-related TCs); of these, TC350 was the most enriched. TC257, TC350, and TC400 were more active in cancer samples from patients with grade 3 disease, triple negative breast cancer, and elevated BMI. Conversely, TC257, TC350, TC371, and TC400 were less active in samples from patients with grade 1 or 2 disease, hormone-positive breast cancer, and low Ki67 score (Fig. 5).

Other DFS-associated TCs were enriched for genes involved in diverse biological processes, including MYC targets and the mitotic spindle. These findings suggest that these TCs capture transcriptional effects from multiple biological processes that may influence the risk of LRR in breast cancer patients.

### DFS-associated TCs showed stability and reproducibility in cross-dataset projection

The 35 DFS-associated TCs were detected across repeated ICA runs with a median of 64 of 96 runs (median credibility index = 0.667; range, 0.250–1.000). In total, 34 of the 35 DFS-associated TCs appeared in at least 25 of the 96 ICA runs; one TC (TC408) appeared in 24 of 96 runs (credibility index = 0.250). Several DFS-associated TCs were recovered in all 96 ICA runs, including TC17, TC37, TC39, TC54, and TC60. These findings support the stability of the DFS-associated transcriptional programs and indicate that they were not dependent on a single ICA initialization (Additional Table 7).

Projection of the 35 DFS-associated TCs into independent TCGA breast carcinoma samples showed that these TCs were detectable across multiple breast cancer subgroups, including triple-negative, ER +/HER2 +, ER +/HER2-, ER-/HER2 +, ER-/HER2-/PR +, and samples with unknown subtype annotation. Several TCs displayed broad activity distributions and outlier activity in TCGA samples, indicating that the transcriptional programs identified in the discovery analysis are also present in an independent patient-derived dataset generated outside the original cohort. In parallel, projection into CCLE breast cancer cell lines showed detectable and outlier activity for multiple DFS-associated TCs, supporting that these transcriptional programs are not restricted to patient bulk tumor profiles but can also be detected in breast cancer cell-line models. Together, these external projections support the reproducibility of the DFS-associated TCs across independent patient data and cell-line systems. We interpret these findings as evidence for reproducibility of TC activity rather than as definitive prospective prognostic validation, which will require future independent cohorts with uniformly collected clinical follow-up data (Additional Fig. 1).

### A subset of 25 DFS-associated TCs were independent of established clinicopathological variables

We evaluated whether the 35 DFS-associated TCs retained an association with DFS after adjustment for intrinsic molecular subtype, tumor grade, and lymph node involvement. In the multivariate Cox models, 25 of the 35 TCs remained associated with DFS at *P* < 0.05, 16 remained associated at *P* < 0.01, and 7 remained associated at P < 0.001. The four adipogenesis-related TCs highlighted in the manuscript remained independently associated with worse DFS after adjustment: TC257 (HR = 15.35, 95% CI 3.70–63.73, *P* = 1.70 × 10^−4), TC350 (HR = 43.36, 95% CI 5.87–320.49, *P* = 2.21 × 10^−4), TC371 (HR = 68.08, 95% CI 7.07–655.12, *P* = 2.59 × 10^−4), and TC400 (HR = 10.28, 95% CI 2.91–36.37, *P* = 2.99 × 10^−4). These results indicate that the prognostic information captured by these TCs is not solely explained by intrinsic subtype, tumor grade, or lymph node involvement. Because the models were fitted to activity scores derived from transcriptomic decomposition, the absolute magnitude of HRs should be interpreted in relation to the scale of the TC activity score; we therefore emphasize the consistency and statistical evidence for independent association rather than the absolute HR magnitude (Additional Table 8).

### Elevated activity of adipogenesis-related TCs in tumor regions of spatially resolved breast cancer transcriptomes and single cell transcriptomes from healthy adipose tissue

**Fig. 6:**
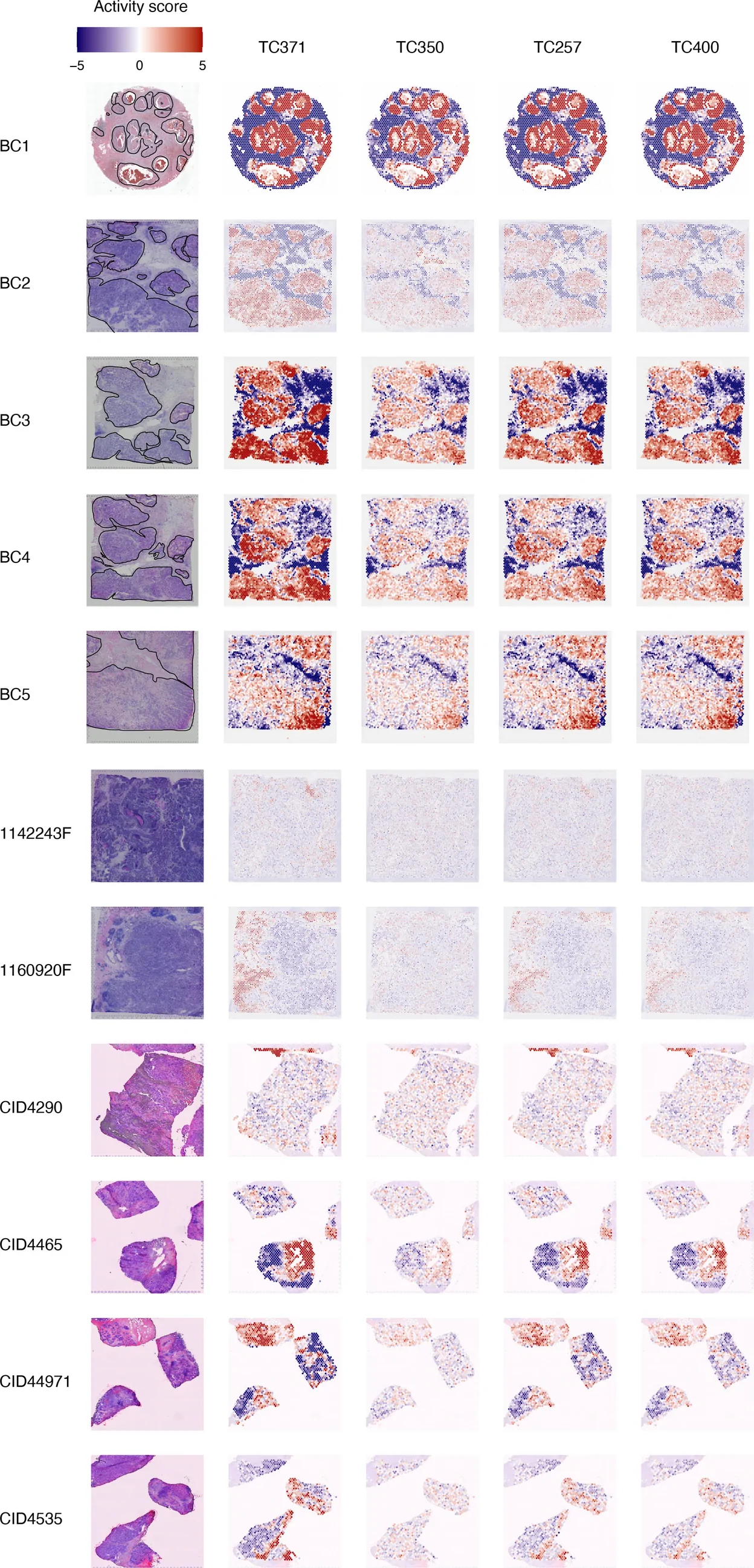
Spatial transcriptomic profiles in breast cancer samples. To pinpoint the areas of significant TC activity in spatial transcriptomic profiles, we employed a permutation-based approach. We ran 5,000 permutations for each TC-profile combination, yielding a p-value that indicates the extent to which the TC's activity in the corresponding profile differs from what would be expected by chance (the null distribution). We then transformed these p-values into z-scores and represented them using a heatmap for 11 samples and four of the TCs related to adipogenesis. The following individual spatial transcriptomic breast cancer samples were used: BC1 (Block 738811QB Sect. 1. Tumor Grade II), BC2(T2N0M0, ER positive, PR negative, Her2 positive, Tumor Grade III), BC3 (Invasive Ductal Carcinoma breast tissue, ER positive, PR negative, Her2 positive), BC4 (Sect. 2 of BC3), BC5 (Invasive Lobular Carcinoma breast tissue, ER positive, PR positive, HER2 negative), CID4290 (BC ER positive), CID4535 (BC ER postive), 1142243 F (BC TNBC), 1160920 F (BC TNBC), CID4465 (BC TNBC) and CID44971 (BC TNBC)

To investigate the co-localized activity of the four adipogenesis-related TCs and determine their cell origin, we analyzed spatial transcriptomic profiles from 11 breast cancer samples and 10,000 single-cell profiles from the adipose tissue of a healthy individual. Analysis of DFS-associated TC activity in the spatial transcriptomes revealed high activity of these four adipogenesis-related TCs in distinct tumor regions (BC1-5; Fig. 6). Likewise, when comparing all DFS-associated TCs in the single-cell data, these four adipogenesis-related TCs consistently showed higher activity than the other TCs (Additional Fig. 2). Notably, they ranked among the top TCs exhibiting significant activity (*p*-value < 0.01) across the highest number of single-cell mRNA expression profiles (Additional Table 9).

## Discussion

Using bulk gene expression profiles from non-metastatic breast tumor samples and human adipose tissue, we identified biological enriched TCs, 35 of which were significantly associated with DFS. Detailed biological characterization of these TCs are available through our public portal.

Among the TCs linked to DFS, four (TC257, TC350, TC371, and TC400) were strongly enrichment for genes involved in adipogenesis, exhibited high active in adipose tissue, and were associated with DFS in breast cancer. The role of adipogenesis in breast cancer was recently investigated using gene expression profiles from 5,098 patients, employing Gene Set Variant Analysis to predict adipogenesis activity based on the Hallmark adipogenesis gene set (Coates et al. 2015). The study showed that heightened adipogenesis activity in TNBC correlates with an unfavorable immune microenvironment, characterized by elevated M2 macrophages infiltration and reduced CD8 + T cells infiltration. Furthermore, in TNBC patients, adipogenesis activity was associated with worse disease specific survival and overall survival, whereas no such correlation was observed in other breast cancer subtypes (Oshi et al. 2021). These findings align with our observation that the four adipogenesis-related TCs were active in patients with TNBC and high-grade disease but not in those with hormone-positive disease, low ki67 score, and intermediate or low-grade disease. However, while the above-mentioned study established a link between adipogenesis activity and an unfavorable immune microenvironment in TNBC, it did not further delve into the underlying transcriptional mechanisms driving this association. Using c-ICA, we were able to identify subtle but biologically relevant transcriptional components within the transcriptomes, allowing a more precise characterization of the transcriptional programs involved in adipogenesis. Moreover, our analysis incorporates adipose tissue from different compartments, providing additional insights into the activity of these TCs, which was not explored in previous studies.

When examining the genes with the highest weight in these four adipogenesis-related TCs, only Fatty Acid Binding Protein 4 (*FABP4*), found in TC350, was described in the gene set ‘adipogenesis’ in Hallmark. *FABP4* is primarily expressed in adipocytes and macrophages, where it regulates metabolic and inflammatory pathways (Storch and Corsico 2008; Zeng et al. 2020). Intracellular FABP4 in macrophages has been identified as a marker of pro-tumor, tumor associated macrophages (TAM). Additionally, elevated circulating FABP4 levels have been linked to breast cancer progression via increased activity of the IL-6/STAT3/ALDH1 pathway, thereby enhancing activity of ALDH1, a recognized stem cell marker in breast cancer (Zeng et al. 2020; Douville et al. 2009; Ginestier et al. 2007; Hancke et al. 2010; Hao et al. 2018). Furthermore, higher circulating FABP4 levels have been associated with an increased risk of breast cancer (Hancke et al. 2010) and elevated FABP4 expression is significantly correlated with shorter DFS and OS in TNBC patients compared to other subtypes (Kim et al. 2015; Guaita-Esteruelas et al. 2018) These findings point to FABP4 as a promising therapeutic target. Indeed, multiple studies have investigated inhibiting FABP4 with small molecule agents and specific antibodies in breast cancer cell lines and animal models (Hao et al. 2024; Cao et al. 2013; Burak et al. 2015; Prentice et al. 2019).

We demonstrated that adipose tissue from different anatomical locations could exhibit distinct transcriptional patterns. Earlier studies also suggested that there might be different transciptional patterns depending on the origin of adipose tissue. For subcutaneous adipose tissue (SAT) and visceral adipose tissue (VAT), it has been shown SAT and VAT differ in both embryogenic origin and in metabolic characteristics (Ibrahim 2010; Vijay et al. 2020; Hwang and Kim 2019). These distinctions could be particularly relevant in reconstructive breast surgery, where adipose tissue from a donor site replaces or augments native breast tissue. We found that adipogenesis-related TC257, TC371, and TC400 generally showed low activity scores in both breast and donor sites; however, TC350 exhibited low activity in breast adipose tissue but high activity in adipose tissue from the abdomen, omentum, and subcutis. Moreover, TC350 was associated with worse DFS, suggesting that adipose tissue from these donor sites could potentially introduce new biological factors that increase LRR. An imaging study of 3,235 women with stage II or III breast cancer showed that increased SAT, but not VAT, correlated with worse overall mortality (Bradshaw et al. 2019) Similar findings have been reported in gastric cancer and hepatocellular carcinoma, where high SAT rather than VAT is a poor prognostic factor (Hessen et al. 2021; Han et al. 2021). In contrast, increased VAT has been associated with higher incidence of colorectal cancer, whereas SAT was not (Vazzana et al. 2012; Im et al. 2018) Taken together, these observations bolster the hypothesis that adipose tissue from different anatomical locations may exert distinct effects on tumor cells. However, these findings should be further investigated prospectively with adipose tissue from different anatomical locations and preferably in a reconstructive setting.

A limitation of our study was the use of retrospectively assembled public datasets and therefore residual study-specific technical variation could not be fully excluded. We therefore explicitly evaluated whether TCs reflected biological signals or study-specific patterns. We initially identified 411 TCs, but only 332 were classified as biologically enriched based on TACNA profiling and/or GSEA. In our study, UMAP visualization showed that the 79 non-enriched TCs exhibited substantially stronger clustering by study ID than the 332 biologically enriched TCs. Quantitatively, clustering by study ID was markedly greater for the non-enriched TCs than for the enriched TCs, and the difference was supported by permutation testing. Therefore, the non-enriched TCs, which likely captured non-biological study or batch effects, were excluded, and all downstream analyses were restricted to the 332 biologically enriched TCs. Although this filtering reduces, residual batch effects, we acknowledge that this cannot be fully eliminated. Future research should focus on assembling independent RNA sequencing cohorts prospectively, to assess generalizability of our findings.

Another limitation was that for the multivariate model, variables such as BMI, BRCA mutation status, and treatment regimen could not be included without avoiding excessive loss of samples and unstable model estimates, due to the retrospective nature of the integrated datasets and incomplete annotation across all studies.

## Conclusion

Our transcriptional mapping of the adipose tissue–breast cancer interfaces reveals distinct patterns associated with LRR, and these patterns may vary in activity depending on the anatomical origin of the adipose tissues. These findings suggest that adipose tissue is not merely a passive bystander in breast cancer, and this might be of particular interest in the context of reconstructive surgery using donor sites from outside the breast. However, future studies should further elucidate how different types of adipose tissue influence tumor biology and clinical outcomes in reconstructive breast surgery.

## Supplementary Information

Below is the link to the electronic supplementary material.

Supplementary file1 (DOCX 18 KB)

Supplementary file2 (DOCX 31 KB)

Supplementary file3 (XLSX 12 KB)

Supplementary file4 (XLSX 21 KB)

Supplementary file5 (XLSX 25 KB)

Supplementary file6 (DOCX 28 KB)

Supplementary file7 (TXT 13 KB)

Supplementary file8 (TXT 6 KB)

Supplementary file9 (XLSX 11 KB)

Supplementary file10 (PNG 1135 KB)

Supplementary file11 (PDF 280 KB)

## Data Availability

The details of the datasets used are provided in the Additional Tables 1 and 2. Comprehensive information on TC composition and GSEA results is available in our public repository: https://transcriptional-landscape-breastcancer-adiposetissue.opendatainscience.net/.

## Abbreviations

LRR: Locoregional recurrences
SVF: Stromal-vascular fraction
CAA: Cancer-associated adipocytes
BRCA!: Breast cancer gene 1
TNB: Triple-negative breast cancer
c-ICA: Consensus-independent component analysis
TC: Transcriptional component
GSEA: Gene Set Enrichment Analysis
DFS: Disease free survival
GEO: Gene Expression Omnibus
ER: Estrogen receptor
PR: Progesterone receptor
HER2: Human epidermal growth factor receptor 2
BMI: Body mass index
MsigDB: Molecular Signatures Database
TACNA: Transcriptional Adaptation to Copy Number Alterations
CAN: Copy number alterations
FDR: False discovery rate
CL: Confidence level
NA: Not available
*FABP4*: Fatty Acid Binding Protein 4
SAT: Subcutaneous adipose tissue
VAT: Visceral adipose tissue

## References

[CR1] Bhattacharya A, Bense RD, Urzúa-Traslaviña CG, de Vries EGE, van Vugt MATM, Fehrmann RSN (2020) Transcriptional effects of copy number alterations in a large set of human cancers. Nat Commun 11(1):715 32024838 10.1038/s41467-020-14605-5 PMC7002723

[CR2] Bhattacharya A, Stutvoet TS, Perla M, Loipfinger S, Jalving M, Reyner AKL et al (2025) Transcriptional pattern enriched for synaptic signaling is associated with shorter survival of patients with high-grade serous ovarian cancer. eLife 13:PR101369 10.7554/eLife.101369 PMC12074640 40359002

[CR3] Bradshaw PT, Cespedes Feliciano EM, Prado CM, Alexeeff S, Albers KB, Chen WY et al (2019) Adipose tissue distribution and survival among women with nonmetastatic breast cancer. Obesity (Silver Spring) 27(6):997–1004 31021535 10.1002/oby.22458 PMC6533153

[CR4] Burak MF, Inouye KE, White A, Lee A, Tuncman G, Calay ES et al (2015) Development of a therapeutic monoclonal antibody that targets secreted fatty acid-binding protein aP2 to treat type 2 diabetes. Sci Transl Med 7(319):319ra205 26702093 10.1126/scitranslmed.aac6336

[CR5] Cao H, Sekiya M, Ertunc ME, Burak MF, Mayers JR, White A et al (2013) Adipocyte lipid chaperone AP2 is a secreted adipokine regulating hepatic glucose production. Cell Metab 17(5):768–778 23663740 10.1016/j.cmet.2013.04.012 PMC3755450

[CR6] Chiappetta P, Roubaud MC, Torrésani B (2004) Blind source separation and the analysis of microarray data. J Comput Biol 11(6):1090–1109 15662200 10.1089/cmb.2004.11.1090

[CR7] Clemons M, Danson S, Hamilton T, Goss P (2001) Locoregionally recurrent breast cancer: incidence, risk factors and survival. Cancer Treat Rev 27(2):67–82 11319846 10.1053/ctrv.2000.0204

[CR8] Clough E, Barrett T (2016) The gene expression omnibus database. Methods Mol Biol 1418:93–110 27008011 10.1007/978-1-4939-3578-9_5 PMC4944384

[CR9] Coates AS, Winer EP, Goldhirsch A, Gelber RD, Gnant M, Piccart-Gebhart M et al (2015) Tailoring therapies–improving the management of early breast cancer: St Gallen International Expert Consensus on the Primary Therapy of Early Breast Cancer 2015. Ann Oncol 26(8):1533–1546 25939896 10.1093/annonc/mdv221 PMC4511219

[CR10] Dirat B, Bochet L, Dabek M, Daviaud D, Dauvillier S, Majed B et al (2011) Cancer-associated adipocytes exhibit an activated phenotype and contribute to breast cancer invasion. Cancer Res 71(7):2455–2465 21459803 10.1158/0008-5472.CAN-10-3323

[CR11] Douville J, Beaulieu R, Balicki D (2009) ALDH1 as a functional marker of cancer stem and progenitor cells. Stem Cells Dev 18(1):17–25 18573038 10.1089/scd.2008.0055

[CR12] Ebrahim NAA, Farghaly TA, Soliman SMA (2025a) Rewiring the transcriptome: diagnostic and therapeutic implications of alternative splicing in solid cancers. Mol Biol Rep 53(1):125 41296088 10.1007/s11033-025-11302-8

[CR13] Ebrahim NAA, Hussein MA, Sobeih ME et al (2025b) From biomarker to targeted therapy: investigating trophoblast cell-surface antigen 2 expression in triple-negative breast cancer– insights from the national cancer institute. BMC Cancer. 25:1008 40474193 10.1186/s12885-025-14402-7 PMC12142858

[CR14] Ebrahim NAA, Amin NH, Hussein MA (2025c) Immunohistochemical profiling of EGFR in triple-negative breast cancer: diagnostic utility and prognostic impact. Indian J Gynecol Oncol. 10.1007/s40944-025-01060-7

[CR15] Ebrahim NAA, Farghaly TA, Soliman SMA (2026) Exosomal blueprint of lung-tropic metastasis: molecular signatures, microenvironmental conditioning, and translational implications in cancer. Med Oncol 43:93 10.1007/s12032-025-03217-y 41452573

[CR16] Fehrmann RS, Karjalainen JM, Krajewska M, Westra HJ, Maloney D, Simeonov A et al (2015) Gene expression analysis identifies global gene dosage sensitivity in cancer. Nat Genet 47(2):115–125 25581432 10.1038/ng.3173

[CR17] Feng LJ, Mauceri K, Berger BE (1994) Autogenous tissue breast reconstruction in the silicone-intolerant patient. Cancer 74(1 Suppl):440–449 8004619 10.1002/cncr.2820741333

[CR18] Ginestier C, Hee Hur M, Charafe-Jauffret E, Monville F, Dutcher J, Brown M et al (2007) ALDGH1 is a marker of normal and malignant human mammary stem cells and a predictor of poor clinical outcome. Cell Stem Cell 1(5):555–567 18371393 10.1016/j.stem.2007.08.014 PMC2423808

[CR19] Guaita-Esteruelas S, Gumà J, Masana L, Borràs J (2018) The peritumoural adipose tissue microenvironment and cancer. The roles of fatty acid binding protein 4 and fatty acid binding protein 5. Mol Cell Endocrinol 462(Pt B):107–118 28163102 10.1016/j.mce.2017.02.002

[CR20] Hammond ME, Hayes DF, Wolff AC, Mangu PB, Temin S (2010) American society of clinical oncology/college of American pathologist’s guideline recommendations for immunohistochemical testing of estrogen and progesterone receptors in breast cancer. J Oncol Pract 6(4):195–197 21037871 10.1200/JOP.777003 PMC2900870

[CR21] Han J, Tang M, Lu C, Shen L, She J, Wu G (2021) Subcutaneous, but not visceral, adipose tissue as a marker for prognosis in gastric cancer patients with cachexia. Clin Nutr 40:5156–5161 34461589 10.1016/j.clnu.2021.08.003

[CR22] Hanahan D, Coussens LM (2012) Accessories to the crime: functions of cells recruited to the tumor microenvironment. Cancer Cell 21(3):309–322 22439926 10.1016/j.ccr.2012.02.022

[CR23] Hanahan D, Weinberg RA (2011) Hallmarks of cancer: the next generation. Cell 144(5):646–674 21376230 10.1016/j.cell.2011.02.013

[CR24] Hancke K, Grubeck D, Hauser N, Kreienberg R, Weiss JM (2010) Adipocyte fatty acid-binding protein as a novel prognostic factor in obese breast cancer pa- tients. Breast Cancer Res Treat 119(2):367–377 19842034 10.1007/s10549-009-0577-9

[CR25] Hancke K, Grubeck D, Hauser N, Kreienberg R, Weiss JM (2010) Adipocyte fatty acid-binding protein as a novel prognostic factor in obese breast cancer patients. Breast Cancer Res Treat 119(2):367–377 19842034 10.1007/s10549-009-0577-9

[CR26] Hao J, Zhang Y, Yan X, Yan F, Sun Y, Zeng J et al (2018) Circulating adipose fatty acid binding protein is a new link underlying obesity-associated breast/mammary tumor development. Cell Metab 28(5):689-705.e5 30100196 10.1016/j.cmet.2018.07.006 PMC6221972

[CR27] Hao J, Jin R, Yi Y, Jiang X, Yu J, Xu Z et al (2024) Development of a humanized anti-FABP4 monoclonal antibody for potential treatment of breast cancer. Breast Cancer Res 26(1):119 39054536 10.1186/s13058-024-01873-y PMC11270797

[CR28] Hwang I, Kim JB (2019) Two faces of White Adipose tissue with heterogeneous adipogenic progenitors. Diabetes Metab J 43:752–762 31902145 10.4093/dmj.2019.0174 PMC6943255

[CR29] Ibrahim MM (2010) Subcutaneous and visceral adipose tissue: structural and functional differences. Obes Rev 11:11–18 19656312 10.1111/j.1467-789X.2009.00623.x

[CR30] Im JP, Kim D, Chung SJ, Jin EH, Han YM, Park MJ et al (2018) Visceral obesity as a risk factor for colorectal adenoma occurrence in surveillance colonoscopy. Gastrointest Endosc 88(1):119-127.e4 29510147 10.1016/j.gie.2018.02.040

[CR31] Kim S, Lee Y, Koo JS (2015) Differential expression of lipid metabolism-related proteins in different breast cancer subtypes. PLoS ONE 10(3):e0119473 25751270 10.1371/journal.pone.0119473 PMC4353724

[CR32] Knapen DG, Hone Lopez S, de Groot DJA, de Haan JJ, de Vries EGE, Dienstmann R et al (2024) Independent transcriptional patterns reveal biological processes associated with disease-free survival in early colorectal cancer. Commun Med (Lond) 4(1):79 38702451 10.1038/s43856-024-00504-z PMC11068726

[CR33] Krastev TK, Jonasse Y, Kon M (2013) Oncological safety of autologous lipoaspirate grafting in breast cancer patients: a systematic review. Ann Surg Oncol 20(1):111–119 22878615 10.1245/s10434-012-2565-2

[CR34] Lohsiriwat V, Curigliano G, Rietjens M, Goldhirsch A, Petit JY (2011) Autologous fat transplantation in patients with breast cancer: “silencing” or “fueling” cancer recurrence? Breast 20(4):351–357 21295982 10.1016/j.breast.2011.01.003

[CR35] Metsalu T, Vilo J (2015) ClustVis: a web tool for visualizing clustering of multivariate data using Principal Component Analysis and heatmap. Nucleic Acids Res 43(W1):W566–W570 25969447 10.1093/nar/gkv468 PMC4489295

[CR36] Oshi M, Tokumaru Y, Angarita FA, Lee L, Yan L, Matsuyama R et al (2021) Adipogenesis in triple-negative breast cancer is associated with unfavorable tumor immune microenvironment and with worse survival. Sci Rep 11(1):12541 34131208 10.1038/s41598-021-91897-7 PMC8206113

[CR37] Ostman A (2012) The tumor microenvironment controls drug sensitivity. Nat Med 18(9):1332–1334 22961158 10.1038/nm.2938

[CR38] Patel NG, Ramakrishnan V (2017) Microsurgical tissue transfer in breast reconstruction. Clin Plast Surg 44(2):345–359 28340667 10.1016/j.cps.2016.12.002

[CR39] Prentice KJ, Saksi J, Hotamisligil GS (2019) Adipokine FABP4 integrates energy stores and counterregulatory metabolic responses. J Lipid Res 60(4):734–740 30705117 10.1194/jlr.S091793 PMC6446704

[CR40] Society AC (2015) Global cancer facts & figures 3rd edition. American Cancer Society, Atlanta

[CR41] Sparano JA, Zhao F, Martino S, Ligibel JA, Perez EA, Saphner T et al (2015) Long-term follow-up of the E1199 phase III trial evaluating the role of taxane and schedule in operable breast cancer. J Clin Oncol 33(21):2353–2360 26077235 10.1200/JCO.2015.60.9271 PMC4500829

[CR42] Storch J, Corsico B (2008) The emerging functions and mechanisms of mammalian fatty acid-binding proteins. Annu Rev Nutr 28:73–95 18435590 10.1146/annurev.nutr.27.061406.093710

[CR43] Tuinder SMH, Beugels J, Lataster A, de Haan MW, Piatkowski A, Saint-Cyr M et al (2018) The lateral thigh perforator flap for autologous breast reconstruction: a prospective analysis of 138 flaps. Plast Reconstr Surg 141(2):257–268 29019861 10.1097/PRS.0000000000004072

[CR44] Urzúa-Traslaviña CG, Leeuwenburgh VC, Bhattacharya A, Loipfinger S, van Vugt MATM, de Vries EGE et al (2021) Improving gene function predictions using independent transcriptional components. Nat Commun 12(1):1464 33674610 10.1038/s41467-021-21671-w PMC7935959

[CR45] van Tienhoven G, Voogd AC, Peterse JL, Nielsen M, Andersen KW, Mignolet F et al (1999) Prognosis after treatment for loco-regional recurrence after mastectomy or breast conserving therapy in two randomised trials (EORTC 10801 and DBCG-82TM). EORTC Breast Cancer Cooperative Group and the Danish Breast Cancer Cooperative Group. Eur J Cancer. 35(1):32–8 10211085 10.1016/s0959-8049(98)00301-3

[CR46] Vazzana N, Riondino S, Toto V, Guadagni F, Roselli M, Davi G et al (2012) Obesity-driven inflammation and colorectal cancer. Curr Med Chem 19:5837–5853 23033947 10.2174/092986712804143349

[CR47] Vijay J, Gauthier MF, Biswell RL, Louiselle DA, Johnston JJ, Cheung WA et al (2020) Single-cell analysis of human adipose tissue identifies depot and disease specific cell types. Nat Metab 2:97–109 32066997 10.1038/s42255-019-0152-6 PMC7025882

[CR48] von Hessen L, Roumet M, Maurer MH, Lange N, Reeves H, Dufour JF et al (2021) High subcutaneous adipose tissue density correlates negatively with survival in patients with hepatocellular carcinoma. Liver Int 41:828–836 33280219 10.1111/liv.14755

[CR49] Voogd AC, van Oost FJ, Rutgers EJ, Elkhuizen PH, van Geel AN, Scheijmans LJ et al (2005) Long-term prognosis of patients with local recurrence after conservative surgery and radiotherapy for early breast cancer. Eur J Cancer 41(17):2637–2644 16115758 10.1016/j.ejca.2005.04.040

[CR50] Wolff AC, Hammond ME, Hicks DG, Dowsett M, McShane LM, Allison KH et al (2013) Recommendations for human epidermal growth factor receptor 2 testing in breast cancer: American Society of Clinical Oncology/College of American Pathologists clinical practice guideline update. J Clin Oncol 31(31):3997–4013 24101045 10.1200/JCO.2013.50.9984

[CR51] Wronska A, Kmiec Z (2012) Structural and biochemical characteristics of various white adipose tissue depots. Acta Physiol (Oxf) 205(2):194–208 22226221 10.1111/j.1748-1716.2012.02409.x

[CR52] Zaha H, Inamine S, Naito T, Nomura H (2006) Laparoscopically harvested omental flap for immediate breast reconstruction. Am J Surg 192(4):556–558 16978975 10.1016/j.amjsurg.2006.06.030

[CR53] Zeng J, Sauter ER, Li B (2020) FABP4: a new player in obesity-associated breast cancer. Trends Mol Med 26(5):437–440 32359475 10.1016/j.molmed.2020.03.004 PMC7646254

[CR54] Zhao R, Kaakati R, Liu X, Xu L, Lee AK, Bachelder R et al (2019) CRISPR/Cas9-mediated BRCA1 knockdown adipose stem cells promote breast cancer progression. Plast Reconstr Surg 143(3):747–756 30817646 10.1097/PRS.0000000000005316 PMC6400326

[CR55] Zhao C, Wu M, Zeng N, Xiong M, Hu W, Lv W et al (2020) Cancer-associated adipocytes: emerging supporters in breast cancer. J Exp Clin Cancer Res 39(1):156 32787888 10.1186/s13046-020-01666-z PMC7425140

[CR56] Zimmerman MP, Pharm BS, Stanton RM (2014) Recurrence of breast cancer years after the initial tumor. Evid-Based Oncol 20:SP14

