# Supplementary material for: Exploring the transcriptional crosstalk between adipose tissue and locoregional recurrence in breast cancer using independent component analysis
Source: Funct Integr Genomics. 2026 Sep 9;26(1):256. doi: 10.1007/s10142-026-02012-w (PMC13553680; doi:10.1007/s10142-026-02012-w)
Supplement: Supplementary file 1 [file 10142_2026_2012_MOESM11_ESM.pdf]

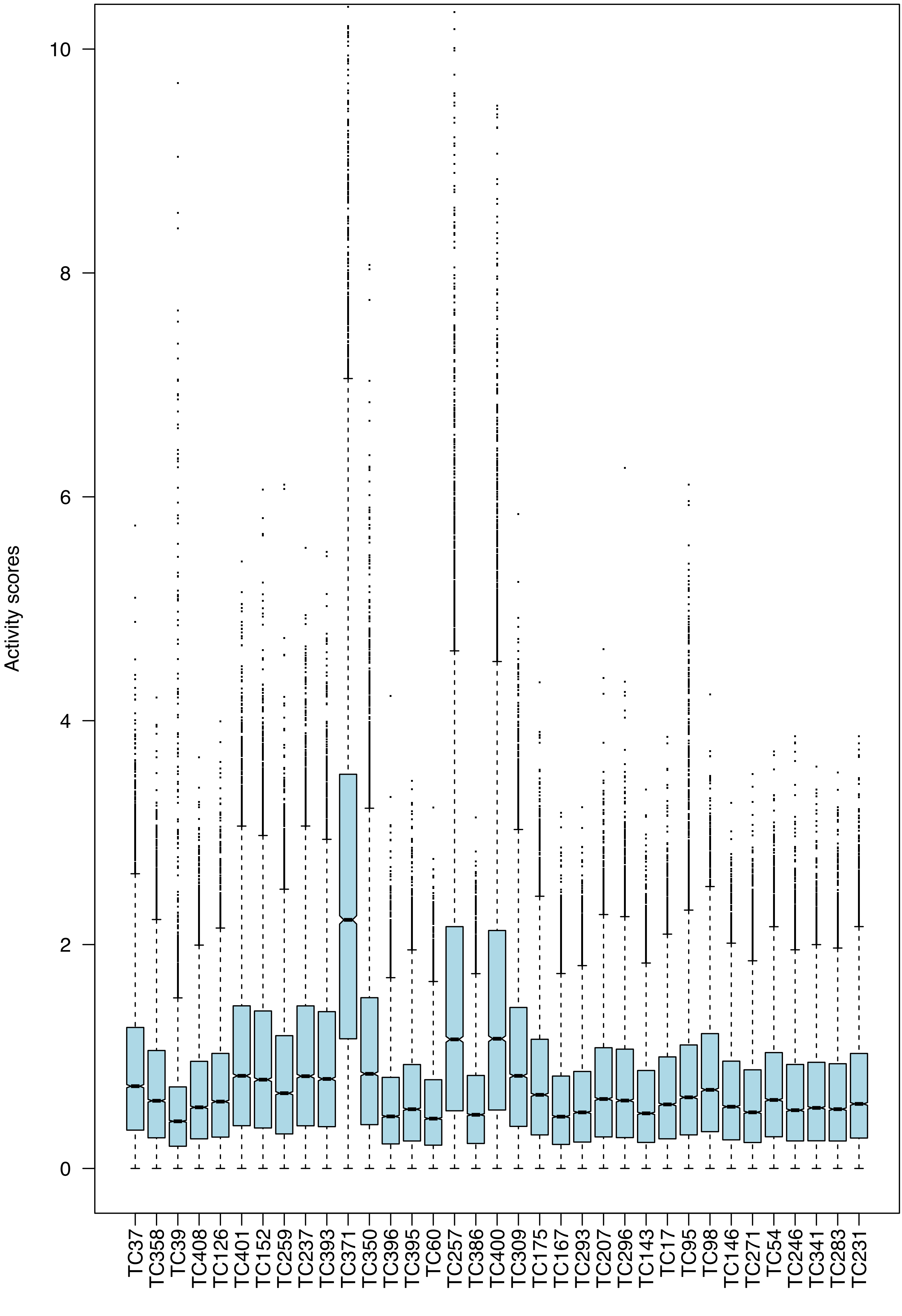

Activity scores
0
2
4
6
8
10
TC37
TC358
TC39
TC408
TC126
TC401
TC152
TC259
TC237
TC393
TC371
TC350
TC396
TC395
TC60
TC257
TC386
TC400
TC309
TC175
TC167
TC293
TC207
TC296
TC143
TC17
TC95
TC98
TC146
TC271
TC54
TC246
TC341
TC283
TC231
