# Supplementary material for: Exploring the transcriptional crosstalk between adipose tissue and locoregional recurrence in breast cancer using independent component analysis
Source: Funct Integr Genomics. 2026 Sep 9;26(1):256. doi: 10.1007/s10142-026-02012-w (PMC13553680; doi:10.1007/s10142-026-02012-w)
Supplement: Supplementary file 2 [file 10142_2026_2012_MOESM1_ESM.docx]

**Supplemental table 1. Overview of studies of the adipose tissue samples**

| **Series** | **Number of Samples** | **PMID** | **Reference** |
| --- | --- | --- | --- |
| GSE3526 | 10 | 16572319 | Roth RB, Hevezi P, Lee J, Willhite D et al. Gene expression analyses reveal molecular relationships among 20 regions of the human CNS. Neurogenetics 2006 May;7(2):67-80. |
| GSE6573 | 2 | 17261642 | Herse F, Dechend R, Harsem NK, Wallukat G et al. Dysregulation of the circulating and tissue-based renin-angiotensin system in preeclampsia. Hypertension 2007 Mar;49(3):604-11. |
| GSE9624 | 11 | 25856673 | Aguilera CM, Gomez-Llorente C, Tofe I, Gil-Campos M et al. Genome-wide expression in visceral adipose tissue from obese prepubertal children. Int J Mol Sci 2015 Apr 8;16(4):7723-37. |
| GSE13070 | 84 | 19841271 | Sears DD, Hsiao G, Hsiao A, Yu JG et al. Mechanisms of human insulin resistance and thiazolidinedione-mediated insulin sensitization. Proc Natl Acad Sci U S A 2009 Nov 3;106(44):18745-50. |
| GSE16615 | 39 | 19689798 | Herrmann J, Rubin D, Häsler R, Helwig U et al. Isomer-specific effects of CLA on gene expression in human adipose tissue depending on PPARgamma2 P12A polymorphism: a double blind, randomized, controlled cross-over study. Lipids Health Dis 2009 Aug 18;8:35. |
| GSE17170 | 70 | 19750004 | Plaisier CL, Horvath S, Huertas-Vazquez A, Cruz-Bautista I et al. A systems genetics approach implicates USF1, FADS3, and other causal candidate genes for familial combined hyperlipidemia. PLoS Genet 2009 Sep;5(9):e1000642. |
| GSE19238 | 2 | 20130649 | Walters RG, Jacquemont S, Valsesia A, de Smith AJ et al. A new highly penetrant form of obesity due to deletions on chromosome 16p11.2. Nature 2010 Feb 4;463(7281):671-5. |
| GSE20950 | 39 | 20678967 | Hardy OT, Perugini RA, Nicoloro SM, Gallagher-Dorval K et al. Body mass index-independent inflammation in omental adipose tissue associated with insulin resistance in morbid obesity. Surg Obes Relat Dis 2011 Jan-Feb;7(1):60-7. |
|  |  | 27900258 | DiStefano MT, Roth Flach RJ, Senol-Cosar O, Danai LV et al. Adipocyte-specific Hypoxia-inducible gene 2 promotes fat deposition and diet-induced insulin resistance. Mol Metab 2016 Dec;5(12):1149-1161. |
|  |  | 28687077 | Saddic LA, Nicoloro SM, Gupta OT, Czech MP et al. Joint analysis of left ventricular expression and circulating plasma levels of Omentin after myocardial ischemia. Cardiovasc Diabetol 2017 Jul 7;16(1):87. |
| GSE26339 | 50 | 22022522 | Lau FH, Deo RC, Mowrer G, Caplin J et al. Pattern specification and immune response transcriptional signatures of pericardial and subcutaneous adipose tissue. PLoS One 2011;6(10):e26092. |
| GSE26457 | 113 | 23057841 | Peri S, de Cicco RL, Santucci-Pereira J, Slifker M et al. Defining the genomic signature of the parous breast. BMC Med Genomics 2012 Oct 11;5:46. |
|  |  | 22025034 | Russo J, Santucci-Pereira J, de Cicco RL, Sheriff F et al. Pregnancy-induced chromatin remodeling in the breast of postmenopausal women. Int J Cancer 2012 Sep 1;131(5):1059-70. |
| GSE26637 | 20 | 22471940 | Soronen J, Laurila PP, Naukkarinen J, Surakka I et al. Adipose tissue gene expression analysis reveals changes in inflammatory, mitochondrial respiratory and lipid metabolic pathways in obese insulin-resistant subjects. BMC Med Genomics 2012 Apr 3;5:9. |
| GSE27657 | 18 | 21125211 | Svensson PA, Jernås M, Sjöholm K, Hoffmann JM et al. Gene expression in human brown adipose tissue. Int J Mol Med 2011 Feb;27(2):227-32. |
|  |  | 23264395 | Nookaew I, Svensson PA, Jacobson P, Jernås M et al. Adipose tissue resting energy expenditure and expression of genes involved in mitochondrial function are higher in women than in men. J Clin Endocrinol Metab 2013 Feb;98(2):E370-8. |
| GSE27916 | 375 | 23264395 | Nookaew I, Svensson PA, Jacobson P, Jernås M et al. Adipose tissue resting energy expenditure and expression of genes involved in mitochondrial function are higher in women than in men. J Clin Endocrinol Metab 2013 Feb;98(2):E370-8. |
|  |  | 23511207 | Mardinoglu A, Agren R, Kampf C, Asplund A et al. Integration of clinical data with a genome-scale metabolic model of the human adipocyte. Mol Syst Biol 2013;9:649. |
|  |  | 24686848 | Falchi M, El-Sayed Moustafa JS, Takousis P, Pesce F et al. Low copy number of the salivary amylase gene predisposes to obesity. Nat Genet 2014 May;46(5):492-7. |
| GSE27949 | 33 | 21426570 | Keller P, Gburcik V, Petrovic N, Gallagher IJ et al. Gene-chip studies of adipogenesis-regulated microRNAs in mouse primary adipocytes and human obesity. BMC Endocr Disord 2011 Mar 22;11:7. |
| GSE28005 | 38 | 22162470 | Alligier M, Meugnier E, Debard C, Lambert-Porcheron S et al. Subcutaneous adipose tissue remodeling during the initial phase of weight gain induced by overfeeding in humans. J Clin Endocrinol Metab 2012 Feb;97(2):E183-92. |
| GSE29410 | 6 | 22286531 | Hoggard N, Cruickshank M, Moar KM, Bashir S et al. Using gene expression to predict differences in the secretome of human omental vs. subcutaneous adipose tissue. Obesity (Silver Spring) 2012 Jun;20(6):1158-67. |
| GSE40231 | 66 | 19997623 | Hägg S, Skogsberg J, Lundström J, Noori P et al. Multi-organ expression profiling uncovers a gene module in coronary artery disease involving transendothelial migration of leukocytes and LIM domain binding 2: the Stockholm Atherosclerosis Gene Expression (STAGE) study. PLoS Genet 2009 Dec;5(12):e1000754. |
| GSE41168 | 70 | - | Yoshino J, Conte C, Fontana L, Mittendorfer B, Imai S, Schechtman KB, Gu CC, Kunz I, Fanelli FR, Patterson BW, Klein S. 2012 |
| GSE71416 | 12 | 26504501 | Doumatey AP, Xu H, Huang H, Trivedi NS et al. Global Gene Expression Profiling in Omental Adipose Tissue of Morbidly Obese Diabetic African Americans. J Endocrinol Metab 2015 Jun;5(3):199-210. |

PMID: Pubmed identification number.
