# Supplementary material for: Exploring the transcriptional crosstalk between adipose tissue and locoregional recurrence in breast cancer using independent component analysis
Source: Funct Integr Genomics. 2026 Sep 9;26(1):256. doi: 10.1007/s10142-026-02012-w (PMC13553680; doi:10.1007/s10142-026-02012-w)
Supplement: Supplementary file 3 [file 10142_2026_2012_MOESM2_ESM.docx]

**Supplemental table 2. Overview of studies of the breast cancer samples**

| **Series** | **Number of samples** | **PMID** | **Reference** |
| --- | --- | --- | --- |
| GSE27120 | 24 | 22235100 | Desmedt C, Majjaj S, Kheddoumi N, Singhal SK et al. Characterization and clinical evaluation of CD10+ stroma cells in the breast cancer microenvironment. Clin Cancer Res 2012 Feb 15;18(4):1004-14. |
| GSE5460 | 127 | 18297396 | Lu X, Lu X, Wang ZC, Iglehart JD et al. Predicting features of breast cancer with gene expression patterns. Breast Cancer Res Treat 2008 Mar;108(2):191-201. |
| GSE5764 | 7 | 17389037 | Turashvili G, Bouchal J, Baumforth K, Wei W et al. Novel markers for differentiation of lobular and ductal invasive breast carcinomas by laser microdissection and microarray analysis. BMC Cancer 2007 Mar 27;7:55. |
| GSE6532 | 87 | 20479250 | Loi S, Haibe-Kains B, Majjaj S, Lallemand F et al. PIK3CA mutations associated with gene signature of low mTORC1 signaling and better outcomes in estrogen receptor-positive breast cancer. Proc Natl Acad Sci U S A 2010 Jun 1;107(22):10208-13. |
|  |  | 17401012 | Loi S, Haibe-Kains B, Desmedt C, Lallemand F et al. Definition of clinically distinct molecular subtypes in estrogen receptor-positive breast carcinomas through genomic grade. J Clin Oncol 2007 Apr 1;25(10):1239-46. |
|  |  | 18498629 | Loi S, Haibe-Kains B, Desmedt C, Wirapati P et al. Predicting prognosis using molecular profiling in estrogen receptor-positive breast cancer treated with tamoxifen. BMC Genomics 2008 May 22;9:239. |
|  |  | 19552798 | Loi S, Sotiriou C, Haibe-Kains B, Lallemand F et al. Gene expression profiling identifies activated growth factor signaling in poor prognosis (Luminal-B) estrogen receptor positive breast cancer. BMC Med Genomics 2009 Jun 24;2:37. |
| GSE7904 | 9 | 16473279 | Richardson AL, Wang ZC, De Nicolo A, Lu X et al. X chromosomal abnormalities in basal-like human breast cancer. Cancer Cell 2006 Feb;9(2):121-32. |
| GSE7515 | 25 | 19666588 | Creighton CJ, Li X, Landis M, Dixon JM et al. Residual breast cancers after conventional therapy display mesenchymal as well as tumor-initiating features. Proc Natl Acad Sci U S A 2009 Aug 18;106(33):13820-5. |
| GSE9195 | 77 | 20479250 | Loi S et al. 2010 Jun |
| GSE10281 | 18 | 19666588 | Creighton CJ, Li X, Landis M, Dixon JM et al. Residual breast cancers after conventional therapy display mesenchymal as well as tumor-initiating features. Proc Natl Acad Sci U S A 2009 Aug 18;106(33):13820-5. |
| GSE10780 | 39 | 19266279 | Chen DT, Nasir A, Culhane A, Venkataramu C et al. Proliferative genes dominate malignancy-risk gene signature in histologically-normal breast tissue. Breast Cancer Res Treat 2010 Jan;119(2):335-46. |
| GSE10810 | 31 | 20029976 | Pedraza V, Gomez-Capilla JA, Escaramis G, Gomez C et al. Gene expression signatures in breast cancer distinguish phenotype characteristics, histologic subtypes, and tumor invasiveness. Cancer 2010 Jan 15;116(2):486-96. |
| GSE11001 | 2 | 21339180 | Budczies J, Weichert W, Noske A, Müller BM et al. Genome-wide gene expression profiling of formalin-fixed paraffin-embedded breast cancer core biopsies using microarrays. J Histochem Cytochem 2011 Feb;59(2):146-57. |
| GSE12276 | 203 | 19421193 | Bos PD, Zhang XH, Nadal C, Shu W et al. Genes that mediate breast cancer metastasis to the brain. Nature 2009 Jun 18;459(7249):1005-9. |
| GSE12790 | 30 | 19567590 | Hoeflich KP, O'Brien C, Boyd Z, Cavet G et al. In vivo antitumor activity of MEK and phosphatidylinositol 3-kinase inhibitors in basal-like breast cancer models. Clin Cancer Res 2009 Jul 15;15(14):4649-64. |
|  |  | 21673316 | Stinson S, Lackner MR, Adai AT, Yu N et al. TRPS1 targeting by miR-221/222 promotes the epithelial-to-mesenchymal transition in breast cancer. Sci Signal 2011 Jun 14;4(177):ra41. |
| GSE16391 | 55 | 19573224 | Desmedt C, Giobbie-Hurder A, Neven P, Paridaens R et al. The Gene expression Grade Index: a potential predictor of relapse for endocrine-treated breast cancer patients in the BIG 1-98 trial. BMC Med Genomics 2009 Jul 2;2:40. |
| GSE16446 | 120 | 21422418 | Desmedt C, Di Leo A, de Azambuja E, Larsimont D et al. Multifactorial approach to predicting resistance to anthracyclines. J Clin Oncol 2011 Apr 20;29(12):1578-86. |
|  |  | 20098429 | Li Y, Zou L, Li Q, Haibe-Kains B et al. Amplification of LAPTM4B and YWHAZ contributes to chemotherapy resistance and recurrence of breast cancer. Nat Med 2010 Feb;16(2):214-8. |
|  |  | 20189874 | Juul N, Szallasi Z, Eklund AC, Li Q et al. Assessment of an RNA interference screen-derived mitotic and ceramide pathway metagene as a predictor of response to neoadjuvant paclitaxel for primary triple-negative breast cancer: a retrospective analysis of five clinical trials. Lancet Oncol 2010 Apr;11(4):358-65. |
|  |  | 26484051 | Haibe-Kains B, Desmedt C, Di Leo A, Azambuja E et al. Genome-wide gene expression profiling to predict resistance to anthracyclines in breast cancer patients. Genom Data 2013 Dec;1:7-10. |
| GSE17907 | 50 | 20932292 | Sircoulomb F, Bekhouche I, Finetti P, Adélaïde J et al. Genome profiling of ERBB2-amplified breast cancers. BMC Cancer 2010 Oct 8;10:539. |
| GSE18728 | 41 | 20012355 | Korde LA, Lusa L, McShane L, Lebowitz PF et al. Gene expression pathway analysis to predict response to neoadjuvant docetaxel and capecitabine for breast cancer. Breast Cancer Res Treat 2010 Feb;119(3):685-99. |
| GSE18864 | 83 | 20100965 | Silver DP, Richardson AL, Eklund AC, Wang ZC et al. Efficacy of neoadjuvant Cisplatin in triple-negative breast cancer. J Clin Oncol 2010 Mar 1;28(7):1145-53. |
|  |  | 20098429 | Li Y, Zou L, Li Q, Haibe-Kains B et al. Amplification of LAPTM4B and YWHAZ contributes to chemotherapy resistance and recurrence of breast cancer. Nat Med 2010 Feb;16(2):214-8. |
|  |  | 20189874 | Juul N, Szallasi Z, Eklund AC, Li Q et al. Assessment of an RNA interference screen-derived mitotic and ceramide pathway metagene as a predictor of response to neoadjuvant paclitaxel for primary triple-negative breast cancer: a retrospective analysis of five clinical trials. Lancet Oncol 2010 Apr;11(4):358-65. |
| GSE19615 | 11 | 20098429 | Li Y, Zou L, Li Q, Haibe-Kains B et al. Amplification of LAPTM4B and YWHAZ contributes to chemotherapy resistance and recurrence of breast cancer. Nat Med 2010 Feb;16(2):214-8 |
| GSE20685 | 327 | 21501481 | Kao KJ, Chang KM, Hsu HC, Huang AT. Correlation of microarray-based breast cancer molecular subtypes and clinical outcomes: implications for treatment optimization. BMC Cancer 2011 Apr 18;11:143. |
| GSE20713 | 88 | 21910250 | Dedeurwaerder S, Desmedt C, Calonne E, Singhal SK et al. DNA methylation profiling reveals a predominant immune component in breast cancers. EMBO Mol Med 2011 Dec;3(12):726-41. |
| GSE21422 | 14 | 21314937 | Kretschmer C, Sterner-Kock A, Siedentopf F, Schoenegg W et al. Identification of early molecular markers for breast cancer. Mol Cancer 2011 Feb 11;10(1):15. |
|  |  | 21468687 | Kretschmer C, Conradi A, Kemmner W, Sterner-Kock A. Latent transforming growth factor binding protein 4 (LTBP4) is downregulated in mouse and human DCIS and mammary carcinomas. Cell Oncol (Dordr) 2011 Oct;34(5):419-34. |
| GSE21653 | 234 | 20490655 | Sabatier R, Finetti P, Cervera N, Lambaudie E et al. A gene expression signature identifies two prognostic subgroups of basal breast cancer. Breast Cancer Res Treat 2011 Apr;126(2):407-20. |
|  |  | 22110708 | Sabatier R, Finetti P, Adelaide J, Guille A et al. Down-regulation of ECRG4, a candidate tumor suppressor gene, in human breast cancer. PLoS One 2011;6(11):e27656. |
| GSE22035 | 43 | 21209903 | Cizkova M, Cizeron-Clairac G, Vacher S, Susini A et al. Gene expression profiling reveals new aspects of PIK3CA mutation in ERalpha-positive breast cancer: major implication of the Wnt signaling pathway. PLoS One 2010 Dec 30;5(12):e15647. |
| GSE22513 | 28 | 20068102 | Bauer JA, Chakravarthy AB, Rosenbluth JM, Mi D et al. Identification of markers of taxane sensitivity using proteomic and genomic analyses of breast tumors from patients receiving neoadjuvant paclitaxel and radiation. Clin Cancer Res 2010 Jan 15;16(2):681-90. |
|  |  | 21633166 | Lehmann BD, Bauer JA, Chen X, Sanders ME et al. Identification of human triple-negative breast cancer subtypes and preclinical models for selection of targeted therapies. J Clin Invest 2011 Jul;121(7):2750-67. |
|  |  | 20878462 | Adams S, Chakravarthy AB, Donach M, Spicer D et al. Preoperative concurrent paclitaxel-radiation in locally advanced breast cancer: pathologic response correlates with five-year overall survival. Breast Cancer Res Treat 2010 Dec;124(3):723-32. |
| GSE22840 | 15 | 20799942 | Hawthorn L, Luce J, Stein L, Rothschild J. Integration of transcript expression, copy number and LOH analysis of infiltrating ductal carcinoma of the breast. BMC Cancer 2010 Aug 27;10:460. |
| GSE23177 | 116 | 21116709 | Smeets A, Daemen A, Vanden Bempt I, Gevaert O et al. Prediction of lymph node involvement in breast cancer from primary tumor tissue using gene expression profiling and miRNAs. Breast Cancer Res Treat 2011 Oct;129(3):767-76. |
| GSE23593 | 50 | 20368555 | Barry WT, Kernagis DN, Dressman HK, Griffis RJ et al. Intratumor heterogeneity and precision of microarray-based predictors of breast cancer biology and clinical outcome. J Clin Oncol 2010 May 1;28(13):2198-206. |
| GSE23720 | 49 | 21339811 | Bekhouche I, Finetti P, Adelaïde J, Ferrari A et al. High-resolution comparative genomic hybridization of inflammatory breast cancer and identification of candidate genes. PLoS One 2011 Feb 9;6(2):e16950. |
|  |  | 22110708 | Sabatier R, Finetti P, Adelaide J, Guille A et al. Down-regulation of ECRG4, a candidate tumor suppressor gene, in human breast cancer. PLoS One 2011;6(11):e27656. |
|  |  | 22666420 | Bertucci F, Lagarde A, Ferrari A, Finetti P et al. 8q24 Cancer risk allele associated with major metastatic risk in inflammatory breast cancer. PLoS One 2012;7(5):e37943. |
| GSE26639 | 226 | 21631949 | de Cremoux P, Valet F, Gentien D, Lehmann-Che J et al. Importance of pre-analytical steps for transcriptome and RT-qPCR analyses in the context of the phase II randomised multicentre trial REMAGUS02 of neoadjuvant chemotherapy in breast cancer patients. BMC Cancer 2011 Jun 1;11:215. |
| GSE54219 | 119 | 26553136 | Massink MP, Kooi IE, Martens JW, Waisfisz Q et al. Genomic profiling of CHEK2*1100delC-mutated breast carcinomas. BMC Cancer 2015 Nov 9;15:877. |
|  |  | 25616998 | Massink MP, Kooi IE, van Mil SE, Jordanova ES et al. Proper genomic profiling of (BRCA1-mutated) basal-like breast carcinomas requires prior removal of tumor infiltrating lymphocytes. Mol Oncol 2015 Apr;9(4):877-88. |
|  |  | 21614566 | Nagel JH, Peeters JK, Smid M, Sieuwerts AM et al. Gene expression profiling assigns CHEK2 1100delC breast cancers to the luminal intrinsic subtypes. Breast Cancer Res Treat 2012 Apr;132(2):439-48. |
| GSE28844 | 32 | 23326553 | Vera-Ramirez L, Sanchez-Rovira P, Ramirez-Tortosa CL, Quiles JL et al. Transcriptional shift identifies a set of genes driving breast cancer chemoresistance. PLoS One 2013;8(1):e53983. |
| GSE28796 | 24 | 21633166 | Lehmann BD, Bauer JA, Chen X, Sanders ME et al. Identification of human triple-negative breast cancer subtypes and preclinical models for selection of targeted therapies. J Clin Invest 2011 Jul;121(7):2750-67. |
| GSE28821 | 42 | 21633166 | Lehmann BD, Bauer JA, Chen X, Sanders ME et al. Identification of human triple-negative breast cancer subtypes and preclinical models for selection of targeted therapies. J Clin Invest 2011 Jul;121(7):2750-67. |
| GSE29044 | 39 | 23704896 | Colak D, Nofal A, Albakheet A, Nirmal M et al. Age-specific gene expression signatures for breast tumors and cross-species conserved potential cancer progression markers in young women. PLoS One 2013;8(5):e63204. |
| GSE29431 | 50 | - | Cuadros M, Cano C, Lopez FJ, Blanco A, Concha A, 2011 |
| GSE29832 | 3 | 22110609 | Gong T, Hartmann N, Kohane IS, Brinkmann V et al. Optimal deconvolution of transcriptional profiling data using quadratic programming with application to complex clinical blood samples. PLoS One 2011;6(11):e27156. |
| GSE31192 | 13 | 23479404 | Harvell DM, Kim J, O'Brien J, Tan AC et al. Genomic signatures of pregnancy-associated breast cancer epithelia and stroma and their regulation by estrogens and progesterone. Horm Cancer 2013 Jun;4(3):140-53. |
| GSE31448 | 24 | 22110708 | Sabatier R, Finetti P, Adelaide J, Guille A et al. Down-regulation of ECRG4, a candidate tumor suppressor gene, in human breast cancer. PLoS One 2011;6(11):e27656. |
| GSE32646 | 101 | 22320227 | Miyake T, Nakayama T, Naoi Y, Yamamoto N et al. GSTP1 expression predicts poor pathological complete response to neoadjuvant chemotherapy in ER-negative breast cancer. Cancer Sci 2012 May;103(5):913-20. |
| GSE33658 | 11 | 21792626 | Massarweh S, Tham YL, Huang J, Sexton K et al. A phase II neoadjuvant trial of anastrozole, fulvestrant, and gefitinib in patients with newly diagnosed estrogen receptor positive breast cancer. Breast Cancer Res Treat 2011 Oct;129(3):819-27. |
| GSE35925 | 14 | 23623780 | Urata YN, Lyra EC, Katayama ML, Basso RA et al. Calcitriol supplementation effects on Ki67 expression and transcriptional profile of breast cancer specimens from post-menopausal patients. Clin Nutr 2014 Feb;33(1):136-42. |
| GSE36774 | 106 | - | Cris Print 2012 |
| GSE40837 | 4 | 24826798 | Massarweh S, Moss J, Wang C, Romond E et al. Impact of adding the multikinase inhibitor sorafenib to endocrine therapy in metastatic estrogen receptor-positive breast cancer. Future Oncol 2014 Dec;10(15):2435-48. |
| GSE42568 | 101 | 23740839 | Clarke C, Madden SF, Doolan P, Aherne ST et al. Correlating transcriptional networks to breast cancer survival: a large-scale coexpression analysis. Carcinogenesis 2013 Oct;34(10):2300-8. |
| GSE43358 | 57 | 25412710 | Fumagalli D, Blanchet-Cohen A, Brown D, Desmedt C et al. Transfer of clinically relevant gene expression signatures in breast cancer: from Affymetrix microarray to Illumina RNA-Sequencing technology. BMC Genomics 2014 Nov 21;15:1008. |
|  |  | 26440892 | Fumagalli D, Gacquer D, Rothé F, Lefort A et al. Principles Governing A-to-I RNA Editing in the Breast Cancer Transcriptome. Cell Rep 2015 Oct 13;13(2):277-89. |
| GSE43365 | 111 | 23990869 | Metzger-Filho O, Catteau A, Michiels S, Buyse M et al. Genomic Grade Index (GGI): feasibility in routine practice and impact on treatment decisions in early breast cancer. PLoS One 2013;8(8):e66848. |
| GSE43502 | 8 | 23549873 | Yu KD, Zhu R, Zhan M, Rodriguez AA et al. Identification of prognosis-relevant subgroups in patients with chemoresistant triple-negative breast cancer. Clin Cancer Res 2013 May 15;19(10):2723-33. |
| GSE45827 | 130 | 27006338 | Gruosso T, Mieulet V, Cardon M, Bourachot B et al. Chronic oxidative stress promotes H2AX protein degradation and enhances chemosensitivity in breast cancer patients. EMBO Mol Med 2016 May;8(5):527-49. |
| GSE46222 | 48 | - | Cho Y, Jin L, Riggins RB, Xuan J, Liu M, Clarke R, 2015 |
| GSE47389 | 47 | 23993096 | Zhang XH, Jin X, Malladi S, Zou Y et al. Selection of bone metastasis seeds by mesenchymal signals in the primary tumor stroma. Cell 2013 Aug 29;154(5):1060-1073. |
| GSE48391 | 78 | 24098497 | Huang CC, Tu SH, Lien HH, Jeng JY et al. Concurrent gene signatures for han chinese breast cancers. PLoS One 2013;8(10):e76421. |
| GSE50567 | 34 | 21196292 | Lisowska KM, Dudaladava V, Jarzab M, Huzarski T et al. BRCA1-related gene signature in breast cancer: the role of ER status and molecular type. Front Biosci (Elite Ed) 2011 Jan 1;3:125-36. |
| GSE52322 | 29 | 16467094 | Dressman HK, Hans C, Bild A, Olson JA et al. Gene expression profiles of multiple breast cancer phenotypes and response to neoadjuvant chemotherapy. Clin Cancer Res 2006 Feb 1;12(3 Pt 1):819-26. |
|  |  | 25811737 | Siamakpour-Reihani S, Owzar K, Jiang C, Scarbrough PM et al. Genomic profiling in locally advanced and inflammatory breast cancer and its link to DCE-MRI and overall survival. Int J Hyperthermia 2015 Jun;31(4):386-95. |
| GSE52327 | 16 | 24511467 | Liu S, Cong Y, Wang D, Sun Y et al. Breast cancer stem cells transition between epithelial and mesenchymal states reflective of their normal counterparts. Stem Cell Reports 2014 Jan 14;2(1):78-91. |
| GSE54002 | 417 | 25214461 | Tan TZ, Miow QH, Miki Y, Noda T et al. Epithelial-mesenchymal transition spectrum quantification and its efficacy in deciphering survival and drug responses of cancer patients. EMBO Mol Med 2014 Oct;6(10):1279-93. |
| GSE58792 | 51 | 25190728 | Shike M, Doane AS, Russo L, Cabal R et al. The effects of soy supplementation on gene expression in breast cancer: a randomized placebo-controlled study. J Natl Cancer Inst 2014 Sep;106(9). PMID: 25190728 |
| GSE103091 | 107 | 25887482 | Jézéquel P, Loussouarn D, Guérin-Charbonnel C, Campion L et al. Gene-expression molecular subtyping of triple-negative breast cancer tumours: importance of immune response. Breast Cancer Res 2015 Mar 20;17:43. |
|  |  | 31101122 | Jézéquel P, Kerdraon O, Hondermarck H, Guérin-Charbonnel C et al. Identification of three subtypes of triple-negative breast cancer with potential therapeutic implications. Breast Cancer Res 2019 May 17;21(1):65. |
| GSE58984 | 94 | - | Fumagalli D, Loi S, Majjaj S, Neven P, Loibl S, Denkert C, Sotiriou C, 2016 |
| GSE61304 | 58 | 26517092 | Grinchuk OV, Motakis E, Yenamandra SP, Ow GS et al. Sense-antisense gene-pairs in breast cancer and associated pathological pathways. Oncotarget 2015 Dec 8;6(39):42197-221. |
|  |  | 26474389 | Aswad L, Yenamandra SP, Ow GS, Grinchuk O et al. Genome and transcriptome delineation of two major oncogenic pathways governing invasive ductal breast cancer development. Oncotarget 2015 Nov 3;6(34):36652-74. |
| GSE65216 | 23 | 25848952 | Maubant S, Tesson B, Maire V, Ye M et al. Transcriptome analysis of Wnt3a-treated triple-negative breast cancer cells. PLoS One 2015;10(4):e0122333. |
|  |  | 23700430 | Maire V, Baldeyron C, Richardson M, Tesson B et al. TTK/hMPS1 is an attractive therapeutic target for triple-negative breast cancer. PLoS One 2013;8(5):e63712. |
|  |  | 23144294 | Maire V, Némati F, Richardson M, Vincent-Salomon A et al. Polo-like kinase 1: a potential therapeutic option in combination with conventional chemotherapy for the management of patients with triple-negative breast cancer. Cancer Res 2013 Jan 15;73(2):813-23. |
| GSE66399 | 84 | 26245675 | Guarneri V, Dieci MV, Frassoldati A, Maiorana A et al. Prospective Biomarker Analysis of the Randomized CHER-LOB Study Evaluating the Dual Anti-HER2 Treatment With Trastuzumab and Lapatinib Plus Chemotherapy as Neoadjuvant Therapy for HER2-Positive Breast Cancer. Oncologist 2015 Sep;20(9):1001-10. |
|  |  | 27484801 | Dieci MV, Prat A, Tagliafico E, Paré L et al. Integrated evaluation of PAM50 subtypes and immune modulation of pCR in HER2-positive breast cancer patients treated with chemotherapy and HER2-targeted agents in the CherLOB trial. Ann Oncol 2016 Oct;27(10):1867-73. |
| GSE70233 | 22 | 17317830 | Harris LN, You F, Schnitt SJ, Witkiewicz A et al. Predictors of resistance to preoperative trastuzumab and vinorelbine for HER2-positive early breast cancer. Clin Cancer Res 2007 Feb 15;13(4):1198-207. |
| GSE71258 | 107 | 26269605 | Xiang J, Hurchla MA, Fontana F, Su X et al. CXCR4 Protein Epitope Mimetic Antagonist POL5551 Disrupts Metastasis and Enhances Chemotherapy Effect in Triple-Negative Breast Cancer. Mol Cancer Ther 2015 Nov;14(11):2473-85. |
| GSE76275 | 264 | 25208879 | Burstein MD, Tsimelzon A, Poage GM, Covington KR et al. Comprehensive genomic analysis identifies novel subtypes and targets of triple-negative breast cancer. Clin Cancer Res 2015 Apr 1;21(7):1688-98. |
|  |  | 26921331 | den Hollander P, Rawls K, Tsimelzon A, Shepherd J et al. Phosphatase PTP4A3 Promotes Triple-Negative Breast Cancer Growth and Predicts Poor Patient Survival. Cancer Res 2016 Apr 1;76(7):1942-53. |
| GSE82173 | 20 | 28522855 | De Marchi T, Timmermans MA, Sieuwerts AM, Smid M et al. Phosphoserine aminotransferase 1 is associated to poor outcome on tamoxifen therapy in recurrent breast cancer. Sci Rep 2017 May 18;7(1):2099. |
|  |  | 32230714 | Sieuwerts AM, Inda MA, Smid M, van Ooijen H et al. ER and PI3K Pathway Activity in Primary ER Positive Breast Cancer Is Associated with Progression-Free Survival of Metastatic Patients under First-Line Tamoxifen. Cancers (Basel) 2020 Mar 27;12(4). |
| GSE87008 | 31 | 28566333 | Raspé E, Coulonval K, Pita JM, Paternot S et al. CDK4 phosphorylation status and a linked gene expression profile predict sensitivity to palbociclib. EMBO Mol Med 2017 Aug;9(8):1052-1066. |
| GSE87377 | 69 | - | Hu Z, Yang B, Chou J, Wang C, Rashid N, Chu Y, Zhou T, Kaufmann W, Wu J, 2016 |
| GSE88770 | 117 | 23028037 | Metzger-Filho O, Michiels S, Bertucci F, Catteau A et al. Genomic grade adds prognostic value in invasive lobular carcinoma. Ann Oncol 2013 Feb;24(2):377-384. |
| GSE90521 | 4 | 28673354 | Brouwers B, Fumagalli D, Brohee S, Hatse S et al. The footprint of the ageing stroma in older patients with breast cancer. Breast Cancer Res 2017 Jul 3;19(1):78. |
| GSE95700 | 57 | 28299476 | Tseng LM, Chiu JH, Liu CY, Tsai YF et al. A comparison of the molecular subtypes of triple-negative breast cancer among non-Asian and Taiwanese women. Breast Cancer Res Treat 2017 Jun;163(2):241-254. |
| GSE102484 | 682 | 28886126 | Cheng SH, Huang TT, Cheng YH, Tan TBK et al. Validation of the 18-gene classifier as a prognostic biomarker of distant metastasis in breast cancer. PLoS One 2017;12(9):e0184372. |
| GSE102907 | 28 | - | Nobuyuki Fukui, 2017 |

PMID: Pubmed identification number
