# Supplementary material for: Exploring the transcriptional crosstalk between adipose tissue and locoregional recurrence in breast cancer using independent component analysis
Source: Funct Integr Genomics. 2026 Sep 9;26(1):256. doi: 10.1007/s10142-026-02012-w (PMC13553680; doi:10.1007/s10142-026-02012-w)
Supplement: Supplementary file 4 [file 10142_2026_2012_MOESM6_ESM.docx]

**Supplemental table 6A. Proportion of samples with significant high activity scores of DFS associated TCs in the different adipose tissue compartments**

| **TC** | **Abdomen** | **Breast** | **Omentum** | **Pericard** | **Parathyroid** | **Subcutaneous** | **Visceral** |
| --- | --- | --- | --- | --- | --- | --- | --- |
| TC37 | 0,000 | 0,000 | 0,073 | 0,000 | 0,000 | 0,000 | 0,000 |
| TC358 | 0,000 | 0,000 | 0,024 | 0,000 | 0,000 | 0,000 | 0,000 |
| TC39 | 0,000 | 0,000 | 0,024 | 0,000 | 0,000 | 0,000 | 0,152 |
| TC408 | 0,000 | 0,142 | 0,024 | 0,000 | 0,000 | 0,000 | 0,000 |
| TC126 | 0,000 | 0,053 | 0,000 | 0,423 | 0,000 | 0,067 | 0,061 |
| TC401 | 0,128 | 0,239 | 0,171 | 0,038 | 0,000 | 0,010 | 0,136 |
| TC152 | 0,515 | 0,000 | 0,512 | 0,923 | 0,778 | 0,295 | 0,318 |
| TC259 | 0,000 | 0,000 | 0,195 | 0,000 | 0,000 | 0,000 | 0,000 |
| TC237 | 0,351 | 0,292 | 0,146 | 0,038 | 0,000 | 0,010 | 0,227 |
| TC393 | 0,221 | 0,195 | 0,146 | 0,038 | 0,000 | 0,010 | 0,076 |
| TC371 | 0,002 | 0,000 | 0,000 | 0,231 | 0,000 | 0,038 | 0,000 |
| TC350 | 0,422 | 0,000 | 0,317 | 0,654 | 0,556 | 0,248 | 0,242 |
| TC396 | 0,003 | 0,018 | 0,366 | 0,077 | 0,000 | 0,000 | 0,152 |
| TC395 | 0,005 | 0,062 | 0,000 | 0,000 | 0,000 | 0,000 | 0,000 |
| TC60 | 0,101 | 0,726 | 0,073 | 0,346 | 0,000 | 0,267 | 0,318 |
| TC257 | 0,021 | 0,000 | 0,049 | 0,346 | 0,111 | 0,076 | 0,000 |
| TC386 | 0,000 | 0,009 | 0,000 | 0,000 | 0,000 | 0,000 | 0,000 |
| TC400 | 0,057 | 0,000 | 0,049 | 0,423 | 0,111 | 0,114 | 0,030 |
| TC309 | 0,748 | 0,000 | 0,463 | 0,077 | 0,889 | 0,124 | 0,348 |
| TC175 | 0,258 | 0,204 | 0,146 | 0,308 | 0,111 | 0,095 | 0,015 |
| TC167 | 0,011 | 0,000 | 0,024 | 0,000 | 0,000 | 0,010 | 0,000 |
| TC293 | 0,000 | 0,000 | 0,000 | 0,000 | 0,000 | 0,000 | 0,000 |
| TC207 | 0,511 | 0,000 | 0,415 | 0,615 | 0,778 | 0,190 | 0,182 |
| TC296 | 0,170 | 0,035 | 0,146 | 0,000 | 0,222 | 0,000 | 0,076 |
| TC143 | 0,005 | 0,088 | 0,000 | 0,000 | 0,000 | 0,019 | 0,030 |
| TC17 | 0,000 | 0,000 | 0,000 | 0,000 | 0,000 | 0,000 | 0,000 |
| TC95 | 0,006 | 0,027 | 0,000 | 0,000 | 0,000 | 0,000 | 0,182 |
| TC98 | 0,828 | 0,000 | 0,659 | 0,462 | 1,000 | 0,362 | 0,318 |
| TC146 | 0,010 | 0,000 | 0,000 | 0,000 | 0,000 | 0,010 | 0,015 |
| TC271 | 0,029 | 0,000 | 0,073 | 0,154 | 0,111 | 0,029 | 0,045 |
| TC54 | 0,015 | 0,354 | 0,000 | 0,115 | 0,000 | 0,029 | 0,015 |
| TC246 | 0,588 | 0,000 | 0,390 | 0,269 | 1,000 | 0,067 | 0,106 |
| TC341 | 0,000 | 0,000 | 0,000 | 0,000 | 0,000 | 0,000 | 0,000 |
| TC283 | 0,049 | 0,009 | 0,341 | 0,077 | 0,000 | 0,048 | 0,030 |
| TC231 | 0,073 | 0,018 | 0,390 | 0,000 | 0,556 | 0,076 | 0,364 |

**Supplemental table 6B. Proportion of samples with significant low activity scores of DFS associated TCs in the different adipose tissue compartments**

| **TC** | **Abdomen** | **Breast** | **Omentum** | **Pericard** | **Parathyroid** | **Subcutaneous** | **Visceral** |
| --- | --- | --- | --- | --- | --- | --- | --- |
| TC37 | -0,558 | -0,088 | -0,195 | -0,038 | -0,667 | -0,143 | -0,364 |
| TC358 | -0,714 | -0,982 | -0,683 | -0,962 | -0,889 | -0,781 | -0,409 |
| TC39 | -0,998 | -1,000 | -0,976 | -1,000 | -1,000 | -1,000 | -0,773 |
| TC408 | -0,843 | -0,009 | -0,439 | -0,808 | -0,889 | -0,486 | -0,515 |
| TC126 | -0,896 | -0,363 | -0,780 | -0,115 | -0,889 | -0,705 | -0,333 |
| TC401 | -0,024 | -0,018 | -0,293 | -0,154 | -0,333 | -0,219 | -0,015 |
| TC152 | -0,209 | -1,000 | -0,415 | -0,038 | 0,000 | -0,552 | -0,485 |
| TC259 | -1,000 | -0,965 | -0,756 | -0,808 | -1,000 | -0,971 | -1,000 |
| TC237 | -0,002 | -0,018 | -0,098 | -0,038 | -0,111 | -0,029 | 0,000 |
| TC393 | -0,003 | -0,018 | -0,268 | -0,115 | -0,333 | -0,076 | 0,000 |
| TC371 | -0,984 | -1,000 | -0,951 | -0,462 | -0,889 | -0,886 | -1,000 |
| TC350 | -0,252 | -0,973 | -0,195 | -0,077 | 0,000 | -0,657 | -0,500 |
| TC396 | -0,597 | -0,301 | -0,073 | -0,462 | -0,444 | -0,800 | -0,152 |
| TC395 | -0,360 | 0,000 | -0,683 | -0,077 | -0,889 | -0,124 | -0,212 |
| TC60 | -0,172 | 0,000 | -0,268 | 0,000 | -0,889 | -0,029 | 0,000 |
| TC257 | -0,794 | -1,000 | -0,829 | -0,115 | -0,444 | -0,771 | -0,894 |
| TC386 | -0,920 | -0,478 | -1,000 | -0,500 | -1,000 | -0,800 | -0,939 |
| TC400 | -0,631 | -1,000 | -0,732 | -0,192 | -0,444 | -0,714 | -0,803 |
| TC309 | -0,089 | -1,000 | 0,000 | -0,269 | 0,000 | -0,114 | -0,121 |
| TC175 | -0,002 | 0,000 | -0,024 | 0,000 | -0,222 | 0,000 | -0,091 |
| TC167 | -0,170 | -0,956 | -0,146 | -0,423 | 0,000 | -0,400 | -0,288 |
| TC293 | -0,336 | -0,239 | -0,268 | -0,692 | -0,111 | -0,257 | -0,106 |
| TC207 | -0,091 | -1,000 | -0,073 | -0,077 | 0,000 | -0,381 | -0,167 |
| TC296 | -0,032 | -0,177 | -0,317 | -0,846 | -0,111 | -0,238 | -0,273 |
| TC143 | -0,817 | 0,000 | -0,780 | 0,000 | -1,000 | -0,162 | -0,303 |
| TC17 | -0,979 | -1,000 | -1,000 | -0,308 | -1,000 | -0,800 | -0,848 |
| TC95 | -0,890 | -0,168 | -0,634 | -0,385 | -0,778 | -0,781 | -0,485 |
| TC98 | -0,005 | -0,106 | 0,000 | 0,000 | 0,000 | 0,000 | 0,000 |
| TC146 | -0,260 | -0,035 | -0,561 | -0,038 | -0,889 | -0,057 | -0,030 |
| TC271 | -0,174 | -0,442 | -0,171 | -0,154 | -0,222 | -0,200 | -0,106 |
| TC54 | -0,786 | 0,000 | -0,707 | -0,038 | -0,889 | -0,181 | -0,212 |
| TC246 | -0,153 | -1,000 | -0,293 | -0,423 | 0,000 | -0,743 | -0,621 |
| TC341 | -0,567 | -0,858 | -0,878 | -0,154 | -0,889 | -0,571 | -0,379 |
| TC283 | -0,188 | -0,593 | -0,024 | -0,192 | 0,000 | -0,714 | -0,167 |
| TC231 | -0,125 | -0,115 | 0,000 | -0,577 | -0,111 | -0,143 | -0,045 |
